# Supplementary material for: Factors associated with the use of long-lasting insecticidal nets in pregnant women and mothers with children under five years of age in Gaza province, Mozambique
Source: PLOS Glob Public Health. 2024 Jan 16;4(1):e0002811. doi: 10.1371/journal.pgph.0002811 (PMC10790986; doi:10.1371/journal.pgph.0002811)
Supplement: S1 Text — (DOCX) [file pgph.0002811.s002.docx]

**Annex 1: Interview guide for pregnant women and mothers with children under five (version 1.0 of June 11, 2021)**

This guide is aimed at **pregnant women and mothers with children under five years of age** who will participate in the interview, to be carried out in health facilities with the objective of assessing the sociodemographic, cultural, behavioural and institutional factors that influence the use of ITNs in pregnant women and mothers with children under 5 years of age in the Districts of Limpopo and Chibuto.

| **Name of interviewer** | **Date of the interview / /2022 Capture location: 1. ANC/MCHC 2. OutPatient clinic** |
| --- | --- |

**District**

**Health Facility: Residence**

**Interview code**

**Sociodemographic data of pregnant women and those with children under five years of age**

**Age**

**Schooling:** 1. None; 2. Primary; 3. Secondary 4. University

**Marital status:** 1. Single; 2. Married; 3. Widow 4. Divorced

**Ocupation:**1. Domestic; 2. Public

servant ; 3. Private employee 4.

Self-employed

# children under 5 years old

| **Pregnant women's perceptions regarding the appropriate use of ITNs for malaria prevention** | |
| --- | --- |
| 1. In your opinion, what is the use of the mosquito net? 2. How useful have mosquito nets been in your home? 3. What are the groups that should deserve a lot of attention in the use of mosquito nets? | |
| **Myths, taboos and beliefs related to the use of mosquito nets** | |
| a) What are the myths/taboos/beliefs associated with mosquito nets in your community? | |
| **Behavioural barriers to the proper use of ITNs** | |
| 1. What makes it difficult for you/someone else to use the mosquito net? 2. What side effects do you get (what is wrong with you) when you use the mosquito net? 3. What is the importance of using mosquito nets? 4. When should you use the mosquito net (seasons of the year)? 5. Tell us about your experience using the mosquito net on the first day (how did you stretch the net)? | |
| **Key messages conveyed by health professionals during the distribution of ITNs** | |
| 1. What did the health professionals say when they were distributing the net in the antenatal consultation? 2. What did the health professionals say when they gave you the mosquito net at home (both in the 2017   and 2020 net distribution campaigns)? | |
| **Facilitating factors in the use of ITNs** | |
| 1. What do you think is good about the mosquito net offer process? 2. What should be improved in the future to facilitate the ITNs distribution process and consistent use? | |
| **Use of mosquito net** | |
| **Did you sleep under the mosquito net yesterday?**   1. Yes 2. No   If not, why? If yes, how did you use the mosquito net? | **Children under 5 years old slept under the mosquito net yesterday?**   1. Yes 2. No   If not, why ? |
