# Supplementary material for: Factors associated with the use of long-lasting insecticidal nets in pregnant women and mothers with children under five years of age in Gaza province, Mozambique
Source: PLOS Glob Public Health. 2024 Jan 16;4(1):e0002811. doi: 10.1371/journal.pgph.0002811 (PMC10790986; doi:10.1371/journal.pgph.0002811)
Supplement: S2 Text — (DOCX) [file pgph.0002811.s003.docx]

Tema: **Percepções das mulheres gravidas em relação uso adequado da REMILD para prevenção da malária**

| Código | Depoimentos |
| --- | --- |
| Opinião sobre o uso da rede mosquiteira | **a rede mosquiteira serve para quando anoitecer esticarmos para nos prevenir da malaria**  **esticamos a rede dentro de casa e não para outras coisas isso faço quando anoitece** |
|  | **eu uso para não ser picado com mosquitos e apanhar malária**  **para mim serve para não ser picado com os mosquitos e não ter malaria** |
|  | **a rede mosquiteira serve para mosquitos para não apanhar malária**  **esticamos a rede dentro de casa para os mosquitos não me picarem** |
|  | **uso para quando for noite dormir dentro da rede para ser conservado dos mosquitos**  **a rede mosquiteira serve para mosquitos para não apanhar malária** |
|  | **Uso para dormir dentro dela e me proteger dos mosquitos**  **a rede mosquiteira para mim serve para esticar e dormir dentro dela e não sermos picados com mosquitos** |
|  | **Uso para esticar e dormir dentro dela e me proteger dos mosquitos**  **a rede mosquiteira é para esticar para não ser picado com mosquitos** |
|  | **a rede mosquiteira eu uso para esticar la dentro para não ter doenças** |
|  | **a rede mosquiteira eu uso para evitar malária**  **a rede mosquiteira eu uso para evitar mosquitos e evitar a doença de malária** |
|  | **eu também uso para que todos os dias dormir dentro da rede mosquiteira para não apanhar malária**  **na minha opinião a rede serve para quando esticarmos não sermos picados com mosquitos e não apanharmos malária** |
|  | **a rede mosquiteira serve para eu não ser picado com mosquitos e não apanhar malária e para não apanhar doenças** |
|  | **para se proteger malaria, para não ser picada com mosquito, só sei que é para não ser atacada com os mosquitos** |
|  | **a rede mosquiteira serve proteger de mosquitos** |
|  | **Bem! Para mim a rede mosquiteira serve para prevenir coiso, a malária e não serve para outras coisas.** |
|  | **ter malaria, serve para não ter malaria, não sei outra coisa** |
|  | **a rede mosquiteira, em norma, vejo importante vejo que ajuda porque assim que tem mosquito se não usar posso apanhar malária e assim devo proteger a minha pele e a pele das crianças. Assim eu lavo a rede, estico, volto a lavar e estendo no sol.** |
|  | **a rede mosquiteira serve para mim, para prevenir a doença de malária e não serve para outras coisas** |
|  | **a rede mosquiteira serve para muitas coisas porque mata mosquitos e se não usarmos a rede vamos ter malária, mas se tivermos a rede, algumas coisas melhoram como qualquer coisa que pode acontecer no nosso organismo.** |
|  | **na minha opinião, a rede mosquiteira, serve para nos proteger da malaria, porque evita ser picado com mosquitos, evitar doenças porque sempre ser picado com mosquitos você pode ter cólera, muitas doenças enfim mas muito muito a malaria** |
|  | **a rede mosquiteira serve para evitar mosquitos e não apanhar malária e também serve para evitar outras doenças** |
|  |  |
|  | **a rede mosquiteira serve para mim, para nos proteger de doenças. É malária nehhhh** |
|  | **uso para dormir e me prevenir da malária** |
|  | **a rede mosquiteira serve para evitar mosquitos e não apanhar malária e também serve para evitar outras doenças** |
|  |  |
| **Por unanimidade as mulheres grávidas residentes na zona rural entrevistadas, afirmaram que a rede mosquiteira serve para protegerem-se dos mosquitos e não apanharem malária e outras doenças que podem estar relacionadas a malária, conforme evidenciam os depoimentos abaixo:**  **“***Uso para dormir dentro dela e me proteger dos mosquitos. a rede mosquiteira para mim serve para esticar e dormir dentro dela e não sermos picados com mosquitos”*  *“Eu também uso para que todos os dias dormir dentro da rede mosquiteira para não apanhar malária. na minha opinião a rede serve para quando esticarmos não sermos picados com mosquitos e não apanharmos malária e outras doenças.”*  *“a rede mosquiteira serve para mosquitos para não apanhar malária”*  **Todas as mulheres grávidas residentes na zona urbana entrevistadas afirmaram que a rede mosquiteira apenas servia pra proteger as pessoas da picada dos mosquitos e consequentemente evitar a malária, conforme mostram os depoimentos abaixo:**  *“A rede mosquiteira, em norma, vejo importante vejo que ajuda porque assim que tem mosquito se não usar posso apanhar malária e assim devo proteger a minha pele e a pele das crianças.”*  *“A rede mosquiteira serve para mim, para prevenir a doença de malária e não serve para outras coisas”*  *“A rede mosquiteira serve para muitas coisas porque mata mosquitos e se não usarmos a rede vamos ter malária, mas se tivermos a rede, algumas coisas melhoram como qualquer coisa que pode acontecer no nosso organismo.”*  *“Na minha opinião, a rede mosquiteira, serve para nos proteger da malaria, porque evita ser picado com mosquitos, evitar doenças porque sempre ser picado com mosquitos você pode ter cólera, muitas doenças enfim mas muito muito a malaria”* | |
| Conhecimento das mães em relação aos grupos que necessitam de mais atenção na prevenção da malária (grupos vulneráveis) | **posso dar crianças e as mulheres gravidas porque a pessoa gravida deve proteger que está dentro para não ser apanhado com malaria e as crianças também para não ficarem doentes** |
|  | **posso começar pelas crianças e pelas mulheres gravidas porque as crianças e as gravidas, quando são picados, podem ficar doentes até não conseguirem levantar para fazer outras coisas** |
|  | **posso dar crianças e os idosos em primeiro lugar** |
|  | as mulheres gravidas devem se proteger dos mosquitos assim como as crianças e os idosos mas na minha opinião todos deviam usar a rede. |
|  | **podemos dar as crianças e mulheres grávidas.** |
|  | **Posso dar as crianças e mulheres grávidas porque são esses que podem ser muito picados com mosquitos** |
| **Posso dar as crianças e mulheres grávidas** |  |
| **Posso dar as crianças e eu que estou grávida porque eu posso passar a doença para a criança que está dentro de mim** |  |
| devemos cuidar mais de todos os grupos mas em especial as mulheres grávidas e as crianças |  |
| **Eu, para mim, dou mais prioridade as mulheres grávidas, ohhhhhhh, para não, não, não, serem picados com mosquitos para evitarem terem aquela doença de malária, mas nós todos deveríamos usar as redes mosquiteiras. E também a mulher grávida deve usar a rede para proteger a si e a criança que está aqui dentro** |  |
| **A rede é mais e deve ser mais usada mesmo para mim assim que estou assim para não apanhar malária e também cuidar deste que está aqui [bebé] para não apanhar malária e outras doenças e as crianças também devem usar muito a rede mosquiteira** |  |
| **A rede é mais e devia ser usada por todos os grupos mas em especial para mulheres grávidas para a criança sair bem e não sair com doenças, quando nascer** |  |
| **Posso priorizar mulheres grávidas e crianças. A criança tem direito de dormir dentro da rede diariamente, e não só a criança, pessoa adulta também porque a doença não escolhe. Estes devem dormi dentro da rede mosquiteira porque eu ser picado com mosquito, assim que estou grávida, eu posso não sentir nada mas a doença pode ir afectar o que está dentro de mim, já, quando sair, sair já com doenças, muitas doenças enfim já core riscos de eu ficar de baixa no hospital.** |  |
| **Posso priorizar mulheres grávidas e crianças antes de nós adultos, as crianças é que são muito complicadas porque não conseguem sacudir os mosquitos mas nós adultos conseguimos. Para as crianças, os mosquitos só vão picar até ficarem cansados, também os mosquitos as vezes picam a nós adultos e depois só vão aumentar nas crianças e aproveitam deixar doenças nas crianças e também ficarem sem sangue.** |  |
| **posso priorizar mulheres gravidas e crianças** |  |
| **Temos que dar mais atenção a mulheres grávidas porque estas, é importante para prevenir a elas assim como as crianças que estão lá dentro. Pode proteger os outros sim mas o mais importante são mulheres grávidas** |  |
| **se me derem poucas redes [suspiro]… posso dar aquelas pessoas que estão gravidas e as crianças menores, nós que somos grandes podemos cobrir as mantas para que os mosquitos não nos apanhem a nossa pele. As crianças não sabem se proteger.** |  |
| **Eu, para mim, dou mais prioridade as mulheres grávidas, ohhhhhhh, para não, não, não, serem picados com mosquitos para evitarem terem aquela doença de malária, mas nós todos deveríamos usar as redes mosquiteiras. E também a mulher grávida deve usar a rede para proteger a si e a criança que está aqui dentro** |  |
| **A rede é mais e deve ser mais usada mesmo para mim assim que estou assim para não apanhar malária e também cuidar deste que está aqui [bebé] para não apanhar malária e outras doenças e as crianças também devem usar muito a rede mosquiteira** |  |
| **A rede é mais e devia ser usada por todos os grupos mas em especial para mulheres grávidas para a criança sair bem e não sair com doenças, quando nascer** |  |
| **Posso priorizar mulheres grávidas e crianças. A criança tem direito de dormir dentro da rede diariamente, e não só a criança, pessoa adulta também porque a doença não escolhe. Estes devem dormi dentro da rede mosquiteira porque eu ser picado com mosquito, assim que estou grávida, eu posso não sentir nada mas a doença pode ir afectar o que está dentro de mim, já, quando sair, sair já com doenças, muitas doenças enfim já core riscos de eu ficar de baixa no hospital.** |  |
| **Posso priorizar mulheres grávidas e crianças antes de nós adultos, as crianças é que são muito complicadas porque não conseguem sacudir os mosquitos mas nós adultos conseguimos. Para as crianças, os mosquitos só vão picar até ficarem cansados, também os mosquitos as vezes picam a nós adultos e depois só vão aumentar nas crianças e aproveitam deixar doenças nas crianças e também ficarem sem sangue.** |  |
| **posso priorizar mulheres gravidas e crianças** |  |
| **Temos que dar mais atenção a mulheres grávidas porque estas, é importante para prevenir a elas assim como as crianças que estão lá dentro. Pode proteger os outros sim mas o mais importante são mulheres grávidas** |  |
| **se me derem poucas redes [suspiro]… posso dar aquelas pessoas que estão gravidas e as crianças menores, nós que somos grandes podemos cobrir as mantas para que os mosquitos não nos apanhem a nossa pele. As crianças não sabem se proteger.** |  |
| **todos devemos dormir dentro da rede mosquiteira e não acho que existe grupo específico para o uso da rede mosquiteira** |  |
| **posso dar mulheres grávidas e crianças** |  |
| **Para mim, mulheres gravidas e mulheres que tem bebé pequeno, aquelas que dão parto, acho eu antes dos 25 anos porque acho que são, é essa idade que faz as pessoas não usarem coiso, não sei se é falta de tempo ou não prestarem atenção ou é não terem sei lá porque a maioria das pessoas que não dormem coiso dentro das redes, são coiso, são as mulheres ou são crianças que dao parto ou tem filhos antes dos 25 anos ou antes dos 18 anos.M** |  |
| **Para mim, mulheres gravidas e mulheres que tem bebé pequeno, aquelas que dão parto, acho eu antes dos 25 anos porque acho que são, é essa idade que faz as pessoas não usarem coiso, não sei se é falta de tempo ou não prestarem atenção ou é não terem sei lá porque a maioria das pessoas que não dormem coiso dentro das redes, são coiso, são as mulheres ou são crianças que dao parto ou tem filhos antes dos 25 anos ou antes dos 18 anos.M** |  |
| **Posso levar cinco dormirem num sitio e cinco noutro sitio. Posso dar crianças, pessoas gravidas e idosos.** |  |
| **kikikikikiki, será que tem grupo para entrar dentro de rede e o outro não entrar? Por mim não há diferença mas se tiver um rede na casa, por mim ia dar criança, ta ver para a criança ser protegida e não ser protegida, vocês grandes podem ser picados mas as crianças não, também, assim que eu estou assim gravida, não posso dormir fora da rede porque a criança pode sair doente e também não posso deixar minha filha dormir fora de rede.** |  |
| **[choro de criança de longe], isso posso priorizar pessoas gravidas, crianças pequenas e idosos.** |  |
| **Metade das mulheres grávidas residentes na zona rural incluídas no estudo, apontaram as mulheres grávidas e crianças como grupos que necessitam de mais atenção para serem prevenidos da malária, como o uso da rede mosquiteira, vide os depoimentos abaixo:**  *“posso começar pelas crianças e pelas mulheres gravidas porque as crianças e as gravidas, quando são picados, podem ficar doentes até não conseguirem levantar para fazer outras coisas”*  *“podemos dar as crianças e mulheres grávidas.”*  *“Posso dar as crianças e mulheres grávidas porque são esses que podem ser muito picados com mosquitos”*  *“Posso dar as crianças e mulheres grávidas”*  **Uma parte das mulheres entrevistadas, aprofundaram um pouco mais afirmando que as mulheres grávidas devem ser mais protegidas para que a mãe não contraia malária e não passe para a criança ainda em gestação.**  *“posso dar crianças e as mulheres gravidas porque a pessoa gravida deve proteger que está dentro para não ser apanhado com malaria e as crianças também para não ficarem doentes”*  *“Posso dar as crianças e eu que estou grávida porque eu posso passar a doença para a criança que está dentro de mim”*  **Algumas mulheres grávidas, apontaram que para além das mulheres grávidas e crianças, todos os outros grupos deviam ser cuidados e protegidos contra a malária.**  *“as mulheres gravidas devem se proteger dos mosquitos assim como as crianças e os idosos mas na minha opinião todos deviam usar a rede.”*  *“devemos cuidar mais de todos os grupos mas em especial as mulheres grávidas e as crianças*”  **Mais da metade das mulheres gravidas residentes na zona urbana entrevistadas referiram que o grupo que mais necessita de atenção para a prevenção da malária usando a rede mosquiteira, são as mulheres grávidas para não poderem passarem a doença para os fetos assim como as crianças menores, conforme mostram os depoimentos abaixo:**  *“Eu, para mim, dou mais prioridade as mulheres grávidas, ohhhhhhh, para não, não, não, serem picados com mosquitos para evitarem terem aquela doença de malária, mas nós todos deveríamos usar as redes mosquiteiras. E também a mulher grávida deve usar a rede para proteger a si e a criança que está aqui dentro”*  *“Posso priorizar mulheres grávidas e crianças. A criança tem direito de dormir dentro da rede diariamente, e não só a criança, pessoa adulta também porque a doença não escolhe. Estes devem dormi dentro da rede mosquiteira porque eu ser picado com mosquito, assim que estou grávida, eu posso não sentir nada mas a doença pode ir afectar o que está dentro de mim, já, quando sair, sair já com doenças, muitas doenças enfim já core riscos de eu ficar de baixa no hospital.”*  *“Se me derem poucas redes [suspiro]… posso dar aquelas pessoas que estão gravidas e as crianças menores, nós que somos grandes podemos cobrir as mantas para que os mosquitos não nos apanhem a nossa pele. As crianças não sabem se proteger.”*  *“Posso priorizar mulheres grávidas e crianças antes de nós adultos, as crianças é que são muito complicadas porque não conseguem sacudir os mosquitos mas nós adultos conseguimos. Para as crianças, os mosquitos só vão picar até ficarem cansados, também os mosquitos as vezes picam a nós adultos e depois só vão aumentar nas crianças e aproveitam deixar doenças nas crianças e também ficarem sem sangue.”*  **Algumas mulheres gravidas entrevistadas na zona urbana referiram que todos os grupos devem ser prioridades na prevencao da malária, dado que tods estao vulneraveis e o mosquito não escolhe a quem deve picar, vide os depoiments abaixo:**  *“kikikikikiki, será que tem grupo para entrar dentro de rede e o outro não entrar? Por mim não há diferença mas se tiver um rede na casa, por mim ia dar criança, ta ver para a criança ser protegida e não ser protegida, vocês grandes podem ser picados mas as crianças não, também, assim que eu estou assim gravida, não posso dormir fora da rede porque a criança pode sair doente e também não posso deixar minha filha dormir fora de rede.”*  *“Todos devemos dormir dentro da rede mosquiteira e não acho que existe grupo específico para o uso da rede mosquiteira”*  *“A rede é mais e devia ser usada por todos os grupos mas em especial para mulheres grávidas para a criança sair bem e não sair com doenças, quando nascer”* | |

Tema: **Mitos, tabus e crenças relacionados ao uso da rede mosquiteira**

| **Código** | **Depoimento** |
| --- | --- |
| Crenças relacionadas ao uso da rede mosquiteira | **só dizem que é proibido usar no viveiro, apenas devemos usar para nos proteger da malária. O governo proíbe** |
|  | **nada não sei, nunca ouvi nada sobre isso** |
|  | **não sei dizer, nunca ouvi algo** |
|  | **não sei dizer, nunca ouvi algo** |
|  | **nunca ouvi nada sobre a rede mosquiteira, para além de saber da importância da rede mosquiteira** |
|  | **nunca ouvi nada sobre a rede mosquiteira** |
|  | **os líderes sempre proíbem que se leve as redes para Bambene (Lagoa) para ir pescar** |
|  | **não dizem nada** |
|  | **não dizem nada ou já me esqueci.** |
|  | **nunca ouvi nada em relação aos mitos, só sei que dizem que a rede tem doenças e não podemos usar mas eu sei que estão a nos enganar e dizem que nós só nos cobrimos para o mosquito não entrar não é porque o mosquito morre com a rede mosquiteira** |
|  | **não posso mentir, não tenho intimidade para conversar sobre isso com outras pessoas, todos nós temos direito de usar a rede mosquiteira** |
|  | **não sei, nunca ouvi nada sobre os mitos em relação a rede mosquiteira** |
|  | **Na ainda não ouvi quase nada sobre o uso das redes mosquiteiras. O que as vezes tenho ouvido é que não posso usar a rede porque a rede causa infecções, ou o que e não aquilo.** |
|  | **não se diz nada de estranho. Sempre usamos bem as redes e não se diz nada nas comunidades sobre mitos** |
|  | **Na comunidade nunca ouvimos informações/mitos sobre o uso das redes mosquiteiras** |
|  | **bom, isso que diz, não se diz nada de mal lá na comunidade, só se diz que não podemos usar a rede para outras coisas como cobrir as saladas, as mangas, as machambas se não o governo vai negar de nos dar mais apoios sobre as redes mosquiteiras. A rede é para cuidar da nossa pele e da pele das crianças.** |
|  | **não nunca ouvi nada** |
|  | **só ouvimos que devemos usar a rede mosquiteira para proteger as crianças e não para proteger hortas** |
|  | **só já ouvi que a rede antes de ser usada devemos lavar porque não sabemos quais são as pessoas que pegaram esta mesma rede antes de nós, ela pode estar suja.** |
|  | **dizem que devemos cuidar da rede mosquiteira, mesmo agora, se te encontrarem, há aquela coisa de que quer semear as vezes salada depois levar a rede mosquiteira pôr ali, isso estão mais a proibir neste momento, dizem que isso não se pode fazer com a rede mosquiteira e aqueles que levam a rede mosquiteira vão a pesca, esses são também proibidos.** |
|  | **não ouvi nada talvez porque acabo de chegar naquela zona** |
|  | **não dizem nada ou já me esqueci.** |
|  | não ouvi nada |
|  |  |
| **Questionadas sobre as crenças negativas da comunidade que podem influenciar no uso da rede mosquiteira, a maioria disse que nunca ouviu algo negativo a respeito da rede mosquiteira, pelo contrario, sempre os líderes comunitários entre outros influentes tem sensibilizado a população a não usar indevidamente as redes, tal como evidenciam os depoimentos abaixo:**  *“os líderes sempre proíbem que se leve as redes para Bambene (Lagoa) para ir pescar”*  *“nunca ouvi nada sobre a rede mosquiteira, para além de saber da importância da rede mosquiteira”*  *“só dizem que é proibido usar no viveiro, apenas devemos usar para nos proteger da malária. O governo proíbe”*  **Algumas mulheres gravidas residentes na zona urbana, disseram que na comunidade as vezes tem pessoas que lhes dizem para não usarem a rede mosquiteira porque esta causa doenças, mas estas, reportaram igualmente não acreditarem no que esses indivíduos dizem, optando mesmo em continuar a usar a rede mosquiteira como mostram os depoimentos:**  *“nunca ouvi nada em relação aos mitos, só sei que dizem que a rede tem doenças e não podemos usar mas eu sei que estão a nos enganar e dizem que nós só nos cobrimos para o mosquito não entrar não é porque o mosquito morre com a rede mosquiteira”*  *“Na ainda não ouvi quase nada sobre o uso das redes mosquiteiras. O que as vezes tenho ouvido é que não posso usar a rede porque a rede causa infecções, ou o que e não aquilo.”*  **Algumas reportaram que, o que tem escutado com frequencia nas cumudades, que eles devem usar a rede mosquiteira de uma forma adequada, evitar levar estas redes a pesca assim como as hortas tanto na machamba assim como em casa:**  *“Bom, isso que diz, não se diz nada de mal lá na comunidade, só se diz que não podemos usar a rede para outras coisas como cobrir as saladas, as mangas, as machambas se não o governo vai negar de nos dar mais apoios sobre as redes mosquiteiras. A rede é para cuidar da nossa pele e da pele das crianças.”*  *“Só ouvimos que devemos usar a rede mosquiteira para proteger as crianças e não para proteger hortas”*  *“Dizem que devemos cuidar da rede mosquiteira, mesmo agora, se te encontrarem, há aquela coisa de que quer semear as vezes salada depois levar a rede mosquiteira pôr ali, isso estão mais a proibir neste momento, dizem que isso não se pode fazer com a rede mosquiteira e aqueles que levam a rede mosquiteira vão a pesca, esses são também proibidos.”* | |

Tema: **Barreiras comportamentais para o uso da rede mosquiteira tratada com insecticida de longa duração**

| **Código** | **Depoimento** |
| --- | --- |
| **Barreiras pessoais e de conhecidos** | **mesmo não aceitando, eu uso para não morrer, a rede só cria alergias quando você dorme por cima da rede, assim pode aquecer. Quando durmo por cima da rede, me cria borbulhas e aquece muito**  **muitas pessoas dizem que não gostam de usar a rede mosquiteira porque ficam com alergias que dificilmente passam e outros dizem que não gostam porque a rede aquece muito e lhes causa borbulhas** |
|  | **nunca senti nada de errado, mesmo agora uso a rede mosquiteira**  **heehhhhhh nunca me disseram nada e quando converso com outras pessoas nunca dizem na minha presença e os meus vizinhos também usam a rede mosquiteira** |
|  | **nunca ouvi nada de estranho a falarem. Já ouvi algumas pessoas a dizerem que a rede lhes causa alergia até saírem borbulhas grande mas só acontece caso use a rede sem lavar.** |
|  | **nunca me aconteceu nada de errado**  **a comunidade diz que algumas pessoas não conseguem usar a rede mosquiteira porque ficam sufocados, sem ar assim como sair borbulhas quando utilizarem a rede mosquiteira e isso acontece quando a pessoa não tratar bem a rede mosquiteira.** |
|  | **Outras pessoas dizem que não conseguem usar a rede mosquiteira porque lhes sufoca quando dormem dentro da rede.** |
|  | **nunca me aconteceu nada**  **nunca ouvi pessoas a dizerem que não gostam da rede mosquiteira.** |
|  | **as pessoas dizem que não gostam de usar a rede mosquiteira porque não tem mosquitos e levam as redes para Bambene para ir pescar** |
|  | **nunca me aconteceu nada porque sempre lavo e estendo para secar**  **nunca encontrei pessoas que não gostam da rede mosquiteira, todas que encontro gostam mas as vezes dizem que se você não lava pode lhe causar irritações na pele** |
|  | **nunca me aconteceu nada, sempre que estou dentro da rede me sinto bem**  **algumas pessoas só deixam de usar as redes mosquiteira e nunca dizem o motivo** |
|  | **Outros dizem que não usam porque não gostam, de outras coisas já não sei**  **Eu nunca tive nenhum incómodo ao usar a rede mosquiteira** |
|  | **nunca me aconteceu nada, sempre que estou dentro da rede me sinto bem e nunca tive problemas**  **nunca ouvi outras pessoas a dizer que tiveram dificuldades** |
|  | **Outras pessoas usam a rede mosquiteira para servir de capoeira para galinhas e patos. Algumas vizinhas dizem que a rede aquece e costuma a causar borbulhas e preferem usar dragão por causa disso**  **Eu, eu, acontece o aquecimento por causa da rede mosquiteira mas uso ventoinha para ultrapassar este problema. E depois disso nunca me aconteceu mais nada** |
|  | **Para mim ainda não me fez nada, sempre me senti a vontade ao usar a rede mosquiteira.**  **isso sempre as mulheres dizem, pior quando a pessoa está grávida as vezes diz a rede mosquiteira lhe faz sair aquilo ali, borbulhas, coisas mais por ali.** |
|  | **Para mim ainda não me fez nada, só já me criou um pouco de calor mas passou.**  **isso das pessoas que não gostam de usar a rede mosquiteira, são aqueles que gostam de beber bebidas, pois as pessoas quando voltam de onde voltam grossas já não tem tempo de esticar a rede mosquiteira, só dormem, se vão picar os mosquitos se não vão picar, ele só vai acordar e sentir muitas dores e perceber que não usou a rede. Também existem outras pessoas que dizem que as redes as vezes não fazem nada e antes também diziam que as redes criam alergias e também ficavam asfixiadas mas isso acontece quando não se lava, se lavar isso reduz esses efeitos.** |
|  | **já escutei algumas pessoas a dizerem que a rede faz mal, lhes traz alergias, não apanham ar e nunca ouvi mais coisas**  **para mim as vezes fico com fobia e também tenho tido dificuldades respiratórias mas depois passa e agora já me acostumei com o uso da rede apesar de que as vezes dentro da rede aquece muito** |
|  | **Para mim nunca aconteceu nada.**  **Algumas? Outras não gostam, porque essa coisa de que [pausa de 7 segundos], estão a dizer que preferem usar dragão para usar porque a rede mosquiteira é incómodo e estão a dizer que não apanham bem o ar e estão a se sentir incomodadas** |
|  | **Muitas pessoas falam mal das redes mosquiteiras, como por exemplo, dizem que não apanham ar, lhes cria borbulhas e preferem não usar a rede mosquiteira e eu sempre lhes convenço que um dia vão se habituar**  **antes de eu saber que a rede devia ser lavada primeiro, uma vez levei a rede sem lavar e aquele remédio que está nas redes me criou alergias e fiquei a respirar mal mas não sabia o que fazer mas também apareceu um irmão e eu lhe disse que não usaria mais a rede mosquiteira porque me criou alergias e ele disse para não usar a rede antes de lavar e deixar arejar no só e fiz isso. Depois disso vejo a rede ajuda muito** |
|  | **Tem aquelas que dizem que as vezes quando estão na rede não respiram bem e não usam a rede mosquiteira. Se cobrirem a rede mosquiteira, transpirarem e não gostarem disso.**  **eu uso a rede mosquiteira para não ser picada com o mosquito, se eu não usar, hei de ter malária. Nunca tive problemas. Assim que me deram a rede vou estender primeiro no sol, para queimar com sol e depois irei usar** |
|  | **Nunca aconteceu nada para mim.**  **para mim não sei como explicar mas posso dizer de que aquilo, é falta de coiso, é as pessoas não saberem se prevenirem, porque se no Hospital dizem para dormirem na rede mosquiteira e não dormirem na rede mosquiteira, aquilo é falta de coiso, bem eu não tenho nada a ver com a minha saúde nehhhh, não se preocupam com a sua saúde. E outras pessoas é não gostarem** |
|  | **tem aquelas que dizem que as vezes quando estão na rede não respiram bem e não usam a rede mosquiteira**  **tenho tido dificuldades de respirar mas mesmo assim uso a rede** |
|  | **sinto muito calor quando estou dentro de rede. E agora fico muito feliz com o uso da rede mosquiteira** |
|  | **isso nunca ouvi nada sobre as dificuldades, as vezes sentimos muito calor e deixarmos a rede por causa disso** |
|  |  |
|  |  |
|  |  |
|  |  |
| **Mais que a metade das mulheres entrevistadas apontaram como suas assim como de outras pessoas barreiras para o uso da rede mosquiteira as seguintes: sufoco, aquecimento e alergias, barreiras estas que também foram apontadas como motivos para o não uso ou uso irregular da REMILD conforme mostram os depoimentos abaixo:**  *“Nesse ponto, no inicio, vou falar, daquelas coisas de borbulhas depois de usar a rede mas, sempre que eu vinha aqui no hospital, cruzava malaria mas agora que já utilizo a rede e já que durmo dentro da rede, já estou bem e já sinto a importância da rede e já não estou com aquelas picadas de mosquitos”*  *“Saia borbulhas mas é porque quando recebia a rede e não deixava a rede panhar o sol, eu usava e pensava que ela estava limpa, já ai, sentia comichão e ai eu perguntei e me disseram que tinha que apanhar ar de sol e depois é que utilizo. tem aquelas que dizem que as vezes quando estão na rede não respiram bem e não usam a rede mosquiteira, tenho tido dificuldades de respirar mas mesmo assim uso a rede Para mim nunca aconteceu nada.”*  *“Algumas? Outras não gostam, porque essa coisa de que [pausa de 7 segundos], estão a dizer que preferem usar dragão para usar porque a rede mosquiteira é incómodo e estão a dizer que não apanham bem o ar e estão a se sentir incomodadas Para mim ainda não me fez nada, só já me criou um pouco de calor mas passou.”*  *“Isso das pessoas que não gostam de usar a rede mosquiteira, são aqueles que gostam de beber bebidas, pois as pessoas quando voltam de onde voltam grossas já não tem tempo de esticar a rede mosquiteira, só dormem, se vão picar os mosquitos se não vão picar, ele só vai acordar e sentir muitas dores e perceber que não usou a rede. Também existem outras pessoas que dizem que as redes as vezes não fazem nada e antes também diziam que as redes criam alergias e também ficavam asfixiadas mas isso acontece quando não se lava, se lavar isso reduz esses efeitos.”* | |
| **Preferências do uso da rede mosquiteira por estacoes do ano** | **pior no tempo de coiso, no tempo de verão porque tem muitos mosquitos e no inverno, os mosquitos ainda tem medo de frio e no verão gostam muito por causa de calor talvez lá em Bilene [local onde fazem machambas]. No inverno não há mosquitos** |
|  | **está frio, está calor eu uso a rede mosquiteira, não tenho tempo** |
|  | **uso mais no verão porque há muitos mosquitos e no inverno não.** |
|  | **em mim bem…bem, posso dizer que é uma coisa que já me habituei porque mesmo viajando, viajo com a rede na pasta.** |
|  | **uso mais a rede no tempo de calor porque é o tempo com muitos mosquitos devido as aguas paradas que encontramos lá nas nossas zonas e quando anoitece os mosquitos aumentam. No inverno uso pouco as redes mosquiteiras porque uso mais a manta para não ser picado com mosquito** |
|  | **uso mais a rede no tempo de calor porque é o tempo com muitos mosquitos. No inverno não uso a rede mosquiteira** |
|  | **uso mais a rede no tempo de calor porque tem muitos mosquitos e no inverno não uso porque não tem mosquitos** |
|  | **uso mais a rede no tempo de calor porque tem muitos mosquitos e no inverno não uso muito porque não tem muitos mosquitos.** |
|  | **Eu uso em todo o momento que em todo momento tem mosquitos mas as vezes no inverno tenho me esquecido de usar a rede. Não uso com frequência no inverno porque não tem muitos mosquitos.** |
|  | **Eu uso principalmente as redes mosquiteiras no verão porque tem muitos mosquitos, no inverno não há mosquitos** |
|  | **hahhhh! Todo tempo, é inverno ou verão, dormimos de baixo da rede mosquiteira, porque na nossa zona tem muitos mosquitos e não tem hora que se diz que esta hora hei de usar a rede mosquiteira ahihi.** |
|  | **eu só tiro a rede para ir lavar, uso todos os dias** |
|  | **eu uso em todo tempo. Estou a usar em todo o tempo porque as vezes em tempos de frio há mosquitos mas tem mosquitos bem no verão mas no inverno tem poucos** |
|  | **eu uso a rede em todo o momento, quando há frio assim como quando há calor** |
|  | **eu uso a rede em todo o momento, quando há frio assim como quando há calor. Em todo momento, no inverno tem, no verão também tem os mosquitos.** |
|  | **a rede mosquiteira não tem inverno e nem tem verão porque todo momento há mosquitos, só que bem neste tempo de frio posso dizer que mosquito não é muito assim mas devemos usar a rede mosquiteira porque muito muito no verão não cobrimos e mosquito também é muito, já não devemos ficar sem a rede mosquiteira** |
|  | **Para mim, todo tempo temos que usar a rede mosquiteira porque mesmo agora que é tempo de frio, tem muitos mosquitos, talvez noutras zonas não tem mosquitos** |
|  | **Eu uso em todo o momento que em todo momento tem mosquitos e podemos apanhar malária** |
|  | **Uso mais a rede mosquiteira no verão, e no inverno não. O ar não sai por causa do frio e isso me provoca desconforto.** |
|  | **yahhhh, mesmo agora que tem frio, tem redes mosquiteiras em casa e utilizamos, e como eu já estendi, ela fica sempre na cama e só tiro para lavar** |
|  | **todo momento eu uso a rede mosquiteira porque tanto quando faz calor assim como frio os mosquitos existem** |
|  | **Todos os dias tem que se usar a rede mosquiteira, sempre usamos a rede mosquiteira, sempre uso.** |
|  |  |
|  |  |
| **Quase todas as mulheres afirmaram que usam a rede mosquiteira com mais frequência no verão porque nesta estacão do ano existem muitos mosquitos e no inverno não tem usado com frequência porque não tem muitos mosquitos e em algum momento nem existem. Abaixo veja os depoimentos:**  *“pior no tempo de coiso, no tempo de verão porque tem muitos mosquitos e no inverno, os mosquitos ainda tem medo de frio e no verão gostam muito por causa de calor talvez lá em Bilene [local onde fazem machambas]. No inverno não há mosquitos”*  *“uso mais no verão porque há muitos mosquitos e no inverno não.”*  *“uso mais a rede no tempo de calor porque é o tempo com muitos mosquitos devido as aguas paradas que encontramos lá nas nossas zonas e quando anoitece os mosquitos aumentam.”*  *“ No inverno uso pouco as redes mosquiteiras porque uso mais a manta para não ser picado com mosquito”*  *“Eu uso em todo o momento que em todo momento tem mosquitos mas as vezes no inverno tenho me esquecido de usar a rede. Não uso com frequência no inverno porque não tem muitos mosquitos.”*  *“uso mais a rede no tempo de calor porque tem muitos mosquitos e no inverno não uso muito porque não tem muitos mosquitos.”*  **Algumas mulheres afirmaram que usam a rede mosquiteira tanto no inverno assim como no verão porque em todas as estacoes do ano tem mosquitos, vide os depoimentos abaixo:**  *“Está frio, está calor eu uso a rede mosquiteira, não tenho tempo”*  *“Em mim bem…bem, posso dizer que é uma coisa que já me habituei porque mesmo viajando, viajo com a rede na pasta e uso em todo o tempo porque os mosquitos não tem calor nem frio.”*  **Em relacao as Preferências do uso da rede mosquiteira por estacoes do ano por parte das mulheres grávida, mais que a metade, referiu que usa a rede mosquiteira em todas as estacoes do ano dado que em todo o momento existem os mosquitos, vide os depoimentos abaixo:**  *“hahhhh! Todo tempo, é inverno ou verão, dormimos de baixo da rede mosquiteira, porque na nossa zona tem muitos mosquitos e não tem hora que se diz que esta hora hei de usar a rede mosquiteira ahihi.”*  *“Eu uso em todo tempo. Estou a usar em todo o tempo porque as vezes em tempos de frio há mosquitos mas tem mosquitos bem no verão mas no inverno tem poucos”*  *“A rede mosquiteira não tem inverno e nem tem verão porque todo momento há mosquitos, só que bem neste tempo de frio posso dizer que mosquito não é muito assim mas devemos usar a rede mosquiteira porque muito muito no verão não cobrimos e mosquito também é muito, já não devemos ficar sem a rede mosquiteira”*  **Algumas mulheres, referiram que usam a rede mosquiteira mais no verao pois é neste período em que tem multos mosquitos?:**  *“Eu uso principalmente as redes mosquiteiras no verão porque tem muitos mosquitos, no inverno não há mosquitos”*  *“Uso mais no tempo de calor porque é neste tempo que tem muitos mosquitos. No inverno não uso a rede todos os dias porque não tem muitos mosquitos”*  *“Uso mais a rede mosquiteira no verão, e no inverno não. O ar não sai por causa do frio e isso me provoca desconforto.”* | |
| **Experiencia do uso da rede mosquiteira no primeiro dia** |  |
|  | **eu não lavei a rede, só levei a rede, estiquei na cama porque dizem que a rede tem remédio, eu nunca tive borbulhas** |
|  | **eu lavei a rede, estendi na sombra de manhã e a tardinha tirei para ir esticar eu durmo não sei das horas** |
|  | **comecei por lavar a rede com agua e OMO e depois estendi no sol durante três dias e depois usei lá dentro de casa.** |
|  | **só lavei e, estendi e usei a rede mosquiteira. Primeiro lavei roupas e depois de lavar a roupa com OMO, mergulhei a rede e estendi no sol para secar.** |
|  | **só abri a rede e estiquei na cama** |
|  | **só abri a rede, lavei e estendi no sol para secar e estiquei na cama** |
|  | **só abri a rede, lavei e estendi no sol para secar e estiquei na cama** |
|  | **só abri a rede, lavei e estendi na sombra para secar e estiquei na cama sempre** |
|  | **no meu primeiro dia de utilizar a rede, no momento que me ofereceram a rede, foi em casa, cheguei desmanchei a rede dentro do plástico, depois lavei estendi, fiquei dois dias é que depois usei. Deixei na sombra, em baixo da mafurreira e tirei no terceiro dia** |
|  | **Já não me lembro, mas tive a rede pela primeira vez aqui no Hospital e quando cheguei em casa lavei, pendurei e levei fui deixar dentro de casa, estendi no sol e já não me lembro do tempo que deixei para secar mas não levei um dia** |
|  | **recebemos a rede mosquiteira, dormimos para não sermos picados com mosquitos, não me aconteceu nada. Estendemos no calor** |
|  | **Para mim, senti alguns incómodos, tipo não estou acostumada não é, a dormir na rede, mas para além disso não aconteceu nada de ruim e a primeira vez que comecei a usar a rede foi quando tive gravidez e tive a revê aqui no Hospital. Quando cheguei em casa, lavei a rede mosquiteira deixei no calor por alguns dias, sim smi** |
|  | **não tenho experiência a revelar. Mas também tive problemas para respirar mas depois informei aos vizinhos e eles disseram que devo abrir a rede e estender ao sol para esses problemas desaparecerem** |
|  | **antes de usar, coloquei agua sem sabão e depois usei. Coloquei durante uma hora de tempo para secar** |
|  | **só estiquei na cama e usei e não tive problemas** |
|  | **quando eu recebi a rede, me disseram que ali tem aqueles pontos de amarrar a rede, tenho que chegar, eu sacudi um pouco a rede depois fui amarrar a rede em 4 paredes, depois estiquei a rede e me senti a vontade** |
|  | **recebi a rede, depois levei para casa, lavei e depois tirei para usar. Estendi as 9h e tirei as 16h** |
|  | **quando cheguei em casa tirei no plástico, estendi na sombra, não no sol para apanhar briza para ficar bem, para sair aquilo que as pessoas dizem que tem incómodo, e quando passou duas horas de tempo eu tirei e não cheguei de lavar** |
|  | **Recebemos a rede mosquiteira, dormimos para não sermos picados com mosquitos, não me aconteceu nada.** |
|  | **no meu primeiro dia de utilizar a rede, no momento que me ofereceram a rede, foi em casa, cheguei desmanchei a rede dentro do plástico, depois lavei estendi, fiquei dois dias é que depois usei. Deixei na sombra, em baixo da mafurreira e tirei no terceiro dia** |
|  | **Já não me lembro, mas tive a rede pela primeira vez aqui no Hospital e quando cheguei em casa lavei, pendurei e levei fui deixar dentro de casa, estendi no sol e já não me lembro do tempo que deixei para secar mas não levei um dia** |
|  | **recebemos a rede mosquiteira, dormimos para não sermos picados com mosquitos, não me aconteceu nada. Estendemos no calor** |
|  | **Para mim, senti alguns incómodos, tipo não estou acostumada não é, a dormir na rede, mas para além disso não aconteceu nada de ruim e a primeira vez que comecei a usar a rede foi quando tive gravidez e tive a revê aqui no Hospital. Quando cheguei em casa, lavei a rede mosquiteira deixei no calor por alguns dias, sim smi** |
| **Algumas mulheres referiram que não chegaram de lavar a rede mosquiteira quando receberam pela primeira vez, apenas abriram do plástico e estenderam na cama para o uso, como mostram os depoimentos abaixo:**  *“eu não lavei a rede, só levei a rede, estiquei na cama, apesar de dizerem que a rede tem remédio, eu nunca tive borbulhas”*  *“só abri a rede e estiquei na cama”*  **Cerca da metade das entrevistadas referiu que pela primeira vez a receber a rede mosquiteira, lavaram com OMO e estenderam ao sol, só depois disso esticaram na cama. Vide os depoimentos abaixo:**  *“só abri a rede, lavei e estendi no sol para secar e estiquei na cama”*  *“só lavei e, estendi e usei a rede mosquiteira. Primeiro lavei roupas e depois de lavar a roupa com OMO, mergulhei a rede e estendi no sol para secar.”*  *“comecei por lavar a rede com agua e OMO e depois estendi no sol durante três dias e depois usei lá dentro de casa.”*  **Uma parte das mulheres entrevistadas mencionou várias vezes que na primeira experiência, abriu rede, estendeu na sombra por algum tempo e a noite esticou na cama para se prevenir dos mosquitos:**  *“eu levei a rede, estendi na sombra de manhã e a tardinha tirei para ir esticar eu durmo não sei das horas”*  *“só abri a rede, estendi na sombra para secar e estiquei na cama sempre”*  **Algumas mulheres grávidas residentes na zona urbana, referiu que como experiência no primeiro dia que recebeu a rede mosquiteira, chegaram em casa, lavaram ou deixaram a rede arejar na sombra por algum tempo e depois usaram para se protegerem dos mosquitos e evitarem com que tivesses problemas com a rede.**  *“antes de usar, coloquei agua sem sabão e depois usei. Coloquei durante uma hora de tempo para secar”*  *“Quando cheguei em casa tirei no plástico, estendi na sombra, não no sol para apanhar briza para ficar bem, para sair aquilo que as pessoas dizem que tem incómodo, e quando passou duas horas de tempo eu tirei e não cheguei de lavar”*  *“No meu primeiro dia de utilizar a rede, no momento que me ofereceram a rede, foi em casa, cheguei desmanchei a rede dentro do plástico, depois lavei estendi, fiquei dois dias é que depois usei. Deixei na sombra, em baixo da mafurreira e tirei no terceiro dia”*  **A maioria das mulheres gravidas entrevistadas referiram que quando receberam a rede pela primeira vez, apenas foram esticar na cama ou na esteira para prontamente usarem e as que deixaram arejar, deixaram no calor:**  *“…a primeira vez que comecei a usar a rede foi quando tive gravidez e tive a revê aqui no Hospital. Quando cheguei em casa, lavei a rede mosquiteira deixei no calor por alguns dias, sim SMI”*  *“Já não me lembro, mas tive a rede pela primeira vez aqui no Hospital e quando cheguei em casa lavei, pendurei e levei fui deixar dentro de casa, estendi no sol e já não me lembro do tempo que deixei para secar mas não levei um dia”*  *“Quando eu recebi a rede, me disseram que ali tem aqueles pontos de amarrar a rede, tenho que chegar, eu sacudi um pouco a rede depois fui amarrar a rede em 4 paredes, depois estiquei a rede e me senti a vontade”*  *“só estiquei na cama e usei e não tive problemas”*  **Algumas mulheres grávidas residentes na zona urbana, referiu que na primeira vez que receberam a rede mosquiteira, sentiram incómodo como problemas respiratórios mas foram informadas que deviam primeiro terem deixado a rede arejar antes do seu uso, vide os comentários abaixo:**  *“… também tive problemas para respirar mas depois informei aos vizinhos e eles disseram que devo abrir a rede e estender ao sol para esses problemas desaparecerem.”*  *“Para mim, senti alguns incómodos, tipo não estou acostumada não é, a dormir na rede, mas para além disso não aconteceu nada de ruim e a primeira vez que comecei a usar a rede foi quando tive gravidez e tive aqui no Hospital.”* | |

**Tema: Mensagens chave veiculadas pelos profissionais de Saúde durante a distribuição das REMILD**

| **Código** | **Depoimento** |
| --- | --- |
| **Mensagens sobre o uso da rede mosquiteira veiculadas pelos profissionais de saúde durante a distribuição da rede na Consulta Pré-Natal** |  |
|  | **disseram que temos que usar para não apanhar malaria** |
|  | **aqui só me deram a rede e não disseram o que eu devia fazer** |
|  | **as enfermeiras apenas nos disseram que é para usarmos para nos proteger dos mosquitos e da malária, não nos falaram mais nada.** |
|  | **as enfermeiras não me disseram nada** |
|  | **Aqui no hospital só me deram a rede mosquiteira e não disseram mais nada** |
|  | **Aqui no hospital não disseram mais nada, só me deram a rede** |
|  | **Aqui no hospital não disseram mais nada, só me deram a rede e disseram para usar** |
|  | **Aqui no hospital disseram que devemos esticar a rede para nos proteger dos mosquitos, os mosquitos tem malária e não disseram mais nada que eu me recorde.** |
|  | **Aqui no hospital não me disseram nada** |
|  | **Disseram que tenho que fazer aquilo alí, deixar na sombra durante algum período** |
|  | **não recebi rede nestas campanhas, eu não estava em casa e na minha casa ninguém estava mas recebi aqui no hospital** |
|  | **os que distribuíram as redes na comunidade disseram que não era para lavar mas sim era para esticar no calor para que o remédio não me possa causar mal** |
|  | **Os que distribuíram as redes na comunidade disseram que as redes devemos cuidar bem delas para mesmo quando recebemos a visita, também podemos dar a rede para não serem picados com mosquitos não é só para mim. Pode não apanhar outras coisas mas a rede deve ter. também disseram-nos que não podemos usar antes de lavar pois os que usaram antes de lavar, acordaram com alergias pois eles foram ditos e não seguiram mas dia seguinte muitas pessoas lavaram as redes e ninguém mais teve alergia.** |
|  | **Disseram que temos que tomar muito cuidado com a rede porque mesmo quando se estragar não devemos levar a rede ou a aquela que quando recebe a rede, guarda e não usa, as vezes leva a rede, dá os pescadores irem pescar com a rede, já a rede é mais louvável e devemos cuidar mais da rede porque a rede nos evita muita coisa. Disseram que demos lavar depois de três meses para tirar as poeiras** |
|  | **não me disseram nada** |
|  | **disseram que devíamos estender a rede no sol para que aquele remédio evapore e não termos comichão** |
|  | **não levamos as redes mosquiteiras porque não estava em casa** |
|  | **do mesmo jeito que falei aqui, disseram que depois de estender usar e me disseram para não usar nas saladas e se fizesse isso ia ser picada com os mosquitos** |
|  | **não levamos as redes mosquiteiras porque não estava em casa** |
|  | **Nunca recebi rede mosquiteira em casa** |
|  | **Disseram que tenho que fazer aquilo alí, deixar na sombra durante algum período**  **não recebi rede nestas campanhas, eu não estava em casa e na minha casa ninguém estava mas recebi aqui no hospital**  **os que distribuíram as redes na comunidade disseram que não era para lavar mas sim era para esticar no calor para que o remédio não me possa causar mal**  **Os que distribuíram as redes na comunidade disseram que as redes devemos cuidar bem delas para mesmo quando recebemos a visita, também podemos dar a rede para não serem picados com mosquitos não é só para mim. Pode não apanhar outras coisas mas a rede deve ter. também disseram-nos que não podemos usar antes de lavar pois os que usaram antes de lavar, acordaram com alergias pois eles foram ditos e não seguiram mas dia seguinte muitas pessoas lavaram as redes e ninguém mais teve alergia.**  **Disseram que temos que tomar muito cuidado com a rede porque mesmo quando se estragar não devemos levar a rede ou a aquela que quando recebe a rede, guarda e não usa, as vezes leva a rede, dá os pescadores irem pescar com a rede, já a rede é mais louvável e devemos cuidar mais da rede porque a rede nos evita muita coisa. Disseram que demos lavar depois de três meses para tirar as poeiras**  **não me disseram nada**  **disseram que devíamos estender a rede no sol para que aquele remédio evapore e não termos comichão**  **não levamos as redes mosquiteiras porque não estava em casa**  **do mesmo jeito que falei aqui, disseram que depois de estender usar e me disseram para não usar nas saladas e se fizesse isso ia ser picada com os mosquitos**  **não levamos as redes mosquiteiras porque não estava em casa**  **Nunca recebi rede mosquiteira em casa**  **disseram que as redes é para usarmos para prevenir dos mosquitos de modo a não termos malária não era para usarmos para cobrir os canteiros e nada mais**  **Não disseram nada só nos deram as redes mosquiteiras**  **nada. Os distribuidores disseram que antes de eu usar a rede mosquiteira devo lavar a rede, deixar apanhar sol para desaparecer o remédio que tinha. Com muito calor, devia deixar a reder, apanhar um bom sol durante um período de dois dias ainda a estender depois de lavar. Disseram me para lavar na bacia, usando omo e colocar na outra bacia** |
|  | **Disseram que tenho que fazer aquilo alí, deixar na sombra durante algum período**  **não recebi rede nestas campanhas, eu não estava em casa e na minha casa ninguém estava mas recebi aqui no hospital**  **os que distribuíram as redes na comunidade disseram que não era para lavar mas sim era para esticar no calor para que o remédio não me possa causar mal**  **Os que distribuíram as redes na comunidade disseram que as redes devemos cuidar bem delas para mesmo quando recebemos a visita, também podemos dar a rede para não serem picados com mosquitos não é só para mim. Pode não apanhar outras coisas mas a rede deve ter. também disseram-nos que não podemos usar antes de lavar pois os que usaram antes de lavar, acordaram com alergias pois eles foram ditos e não seguiram mas dia seguinte muitas pessoas lavaram as redes e ninguém mais teve alergia.**  **Disseram que temos que tomar muito cuidado com a rede porque mesmo quando se estragar não devemos levar a rede ou a aquela que quando recebe a rede, guarda e não usa, as vezes leva a rede, dá os pescadores irem pescar com a rede, já a rede é mais louvável e devemos cuidar mais da rede porque a rede nos evita muita coisa. Disseram que demos lavar depois de três meses para tirar as poeiras**  **não me disseram nada**  **disseram que devíamos estender a rede no sol para que aquele remédio evapore e não termos comichão**  **não levamos as redes mosquiteiras porque não estava em casa**  **do mesmo jeito que falei aqui, disseram que depois de estender usar e me disseram para não usar nas saladas e se fizesse isso ia ser picada com os mosquitos**  **não levamos as redes mosquiteiras porque não estava em casa**  **Nunca recebi rede mosquiteira em casa**  **disseram que as redes é para usarmos para prevenir dos mosquitos de modo a não termos malária não era para usarmos para cobrir os canteiros e nada mais**  **Não disseram nada só nos deram as redes mosquiteiras**  **nada. Os distribuidores disseram que antes de eu usar a rede mosquiteira devo lavar a rede, deixar apanhar sol para desaparecer o remédio que tinha. Com muito calor, devia deixar a reder, apanhar um bom sol durante um período de dois dias ainda a estender depois de lavar. Disseram me para lavar na bacia, usando omo e colocar na outra bacia** |
|  | **Disseram que tenho que fazer aquilo alí, deixar na sombra durante algum período**  **não recebi rede nestas campanhas, eu não estava em casa e na minha casa ninguém estava mas recebi aqui no hospital**  **os que distribuíram as redes na comunidade disseram que não era para lavar mas sim era para esticar no calor para que o remédio não me possa causar mal**  **Os que distribuíram as redes na comunidade disseram que as redes devemos cuidar bem delas para mesmo quando recebemos a visita, também podemos dar a rede para não serem picados com mosquitos não é só para mim. Pode não apanhar outras coisas mas a rede deve ter. também disseram-nos que não podemos usar antes de lavar pois os que usaram antes de lavar, acordaram com alergias pois eles foram ditos e não seguiram mas dia seguinte muitas pessoas lavaram as redes e ninguém mais teve alergia.**  **Disseram que temos que tomar muito cuidado com a rede porque mesmo quando se estragar não devemos levar a rede ou a aquela que quando recebe a rede, guarda e não usa, as vezes leva a rede, dá os pescadores irem pescar com a rede, já a rede é mais louvável e devemos cuidar mais da rede porque a rede nos evita muita coisa. Disseram que demos lavar depois de três meses para tirar as poeiras**  **não me disseram nada**  **disseram que devíamos estender a rede no sol para que aquele remédio evapore e não termos comichão**  **não levamos as redes mosquiteiras porque não estava em casa**  **do mesmo jeito que falei aqui, disseram que depois de estender usar e me disseram para não usar nas saladas e se fizesse isso ia ser picada com os mosquitos**  **não levamos as redes mosquiteiras porque não estava em casa**  **Nunca recebi rede mosquiteira em casa**  **disseram que as redes é para usarmos para prevenir dos mosquitos de modo a não termos malária não era para usarmos para cobrir os canteiros e nada mais**  **Não disseram nada só nos deram as redes mosquiteiras**  **nada. Os distribuidores disseram que antes de eu usar a rede mosquiteira devo lavar a rede, deixar apanhar sol para desaparecer o remédio que tinha. Com muito calor, devia deixar a reder, apanhar um bom sol durante um período de dois dias ainda a estender depois de lavar. Disseram me para lavar na bacia, usando omo e colocar na outra bacia** |
|  | **Disseram que tenho que fazer aquilo alí, deixar na sombra durante algum período**  **não recebi rede nestas campanhas, eu não estava em casa e na minha casa ninguém estava mas recebi aqui no hospital**  **os que distribuíram as redes na comunidade disseram que não era para lavar mas sim era para esticar no calor para que o remédio não me possa causar mal**  **Os que distribuíram as redes na comunidade disseram que as redes devemos cuidar bem delas para mesmo quando recebemos a visita, também podemos dar a rede para não serem picados com mosquitos não é só para mim. Pode não apanhar outras coisas mas a rede deve ter. também disseram-nos que não podemos usar antes de lavar pois os que usaram antes de lavar, acordaram com alergias pois eles foram ditos e não seguiram mas dia seguinte muitas pessoas lavaram as redes e ninguém mais teve alergia.**  **Disseram que temos que tomar muito cuidado com a rede porque mesmo quando se estragar não devemos levar a rede ou a aquela que quando recebe a rede, guarda e não usa, as vezes leva a rede, dá os pescadores irem pescar com a rede, já a rede é mais louvável e devemos cuidar mais da rede porque a rede nos evita muita coisa. Disseram que demos lavar depois de três meses para tirar as poeiras**  **não me disseram nada**  **disseram que devíamos estender a rede no sol para que aquele remédio evapore e não termos comichão**  **não levamos as redes mosquiteiras porque não estava em casa**  **do mesmo jeito que falei aqui, disseram que depois de estender usar e me disseram para não usar nas saladas e se fizesse isso ia ser picada com os mosquitos**  **não levamos as redes mosquiteiras porque não estava em casa**  **Nunca recebi rede mosquiteira em casa**  **disseram que as redes é para usarmos para prevenir dos mosquitos de modo a não termos malária não era para usarmos para cobrir os canteiros e nada mais**  **Não disseram nada só nos deram as redes mosquiteiras**  **nada. Os distribuidores disseram que antes de eu usar a rede mosquiteira devo lavar a rede, deixar apanhar sol para desaparecer o remédio que tinha. Com muito calor, devia deixar a reder, apanhar um bom sol durante um período de dois dias ainda a estender depois de lavar. Disseram me para lavar na bacia, usando omo e colocar na outra bacia** |
| **Em relação as mensagens sobre o uso da rede mosquiteira veiculadas pelos profissionais de saúde durante a distribuição da rede na Consulta Pré-Natal, por unanimidade as mulheres entrevistadas referiram apenas foram ditas para usarem a rede mosquiteira para prevenirem-se da malária, nada foram ditas sobre como usar e que cuidados deviam ter com a rede, vide os depoimentos abaixo:**  *“disseram que temos que usar para não apanhar malaria”*  *“Aqui no hospital não disseram mais nada, só me deram a rede e disseram para usar”*  *“Aqui no hospital só me deram a rede mosquiteira e não disseram mais nada”*  *“As enfermeiras apenas nos disseram que é para usarmos para nos proteger dos mosquitos e da malária, não nos falaram mais nada.”*  *“Aqui no hospital disseram que devemos esticar a rede para nos proteger dos mosquitos, os mosquitos tem malária e não disseram mais nada que eu me recorde.”*  **Mais que a metade das mulheres gravidas residentes na zona urbana entrevistadas, referiram que depois de serem fornecidas a rede na consulta pré-natal, apenas foram informadas para usarem a rede para se protegerem das picadas dos mosquitos e consequentemente prevenirem-se da malária assim como protegerem as crianças que estavam a espera, como deviam proceder antes do uso não foram informadas, como ilustram os depoimentos abaixo:**  *“Aqui no hospital disseram que devo usar a rede mosquiteira para me prevenir de doenças não me explicaram mais nada”*  *“As enfermeiras disseram que é para eu usar a rede para me prevenir da malária e disseram que tinha que estender na esteira e me proteger dos mosquitos”*  *“Aqui no hospital disseram que devo esticar no quarto e dormir dentro dele”*  *“Aqui no hospital praticamente as enfermeiras não falaram nada, só me deram a rede e disseram para ir usar”*  *“Disseram que é para a rede mosquiteira ajudar a mim e a criança tanto agora que está na barriga assim como depois de nascer. Se vai ficar doente a criança, deve sempre dormir na rede mosquiteira.”*  *“Disseram que temos que usar para não apanhar malaria e este que está aqui dentro não apanhar doenças por ser picado com mosquitos. Só disseram que devemos esticar na cama ou na esteira”*  **Algumas mulheres gravidas referiram que as enfermeiras questionaram-lhes se já tinham antes recebido a rede mosquiteira e elas responderam que já e estas (as enfermeiras), disseram para fazerem o mesmo que fazem com as redes que receberam antes, como evidenciam os depoimentos abaixo:**  *“Aqui no hospital disseram que deveria lavar antes de lavar. Primeiro perguntaram se eu já usava a rede e eu disse sim e depois me deram a rede mosquiteira alegando que devia fazer o mesmo que faço com a outra rede.”*  *“Já faz tempo e já não me lembro, faz 4 anos mas agora que me deram outra, só me ofereceram não me disseram nada, só disseram que* *porque uma vez que souberam que não é a primeira gravidez não era necessário me explicarem, só me disseram que tenho que dormir na rede porque é importante”*  **Algumas mulheres gravidas foram informadas que antes de usarem a rede mosquiteira, deviam primeiro lavar com OMO e estenderem no sol durante algum tempo, como mostram os depoimentos abaixo:**  *“disseram que é para eu levar a rede mosquiteira estender no sol hoje, depois de eu levar a rede estender no sol hoje, depois queimar com sol, depois levar esta rede me cobrir para não apanhar malária porque se eu não usar a rede mesmo a criança que estou a espera vai sair com malária”*  *“as enfermeiras disseram que devia lavar com sabão, OMO, e estender no sol até secar.”*  *“as enfermeiras disseram que devia chegar em casa estender no sol e depois esticar para me proteger da malária, dormir sempre dentro da rede mosquiteira. Disseram que devia lavar só com água e depois estender no sol para secar”* | |
| **Mensagens sobre o uso da rede mosquiteira veiculadas pelos profissionais de saúde durante a distribuição massiva da rede mosquiteira** |  |
|  | **disseram que devemos usar a rede para nos prevenir a malária e não disseram mais nada** |
|  | **disseram que não querem ver as redes esticadas para proteger as hortas, faz de conta estás a plantar hortas e também não querem ver usarmos as redes levarmos para pescar no *massacatine* [lagoa] e não disseram mais nada** |
|  | **nunca me encontraram na comunidade** |
|  | **Só nos disseram que se você estiver muito apressado, pode lavar a rede mosquiteira com bingo e estender e também pode estender no sol durante três dias** |
|  | **não apanhei em casa** |
|  | **não apanhei em casa, talvez a minha mãe.** |
|  | **só me deram a rede e não disseram mais nada** |
|  | **disseram que devemos lavar com OMO antes de usarmos para evitar que o remédio que está na rede nos cause problemas e que também podíamos estender no sol** |
|  | **disseram que devemos lavar com OMO e sabão bingo antes de usarmos para evitar que o remédio que está na rede nos cause problemas e que também podíamos estender no sol** |
|  | **não levamos as redes mosquiteiras porque não estava em casa** |
|  | **do mesmo jeito que falei aqui, disseram que depois de estender usar e me disseram para não usar nas saladas e se fizesse isso ia ser picada com os mosquitos** |
|  | **não levamos as redes mosquiteiras porque não estava em casa** |
|  | **Nunca recebi rede mosquiteira em casa** |
|  | **disseram que as redes é para usarmos para prevenir dos mosquitos de modo a não termos malária não era para usarmos para cobrir os canteiros e nada mais** |
|  | **Não disseram nada só nos deram as redes mosquiteiras** |
|  | **nada. Os distribuidores disseram que antes de eu usar a rede mosquiteira devo lavar a rede, deixar apanhar sol para desaparecer o remédio que tinha. Com muito calor, devia deixar a reder, apanhar um bom sol durante um período de dois dias ainda a estender depois de lavar. Disseram me para lavar na bacia, usando omo e colocar na outra bacia** |
|  | **não recebi rede nestas campanhas, eu não estava em casa e na minha casa ninguém estava mas recebi aqui no hospital** |
|  | **os que distribuíram as redes na comunidade disseram que não era para lavar mas sim era para esticar no calor para que o remédio não me possa causar mal** |
|  | **Disseram que tenho que fazer aquilo alí, deixar na sombra durante algum período** |
|  | **Disseram que temos que tomar muito cuidado com a rede porque mesmo quando se estragar não devemos levar a rede ou a aquela que quando recebe a rede, guarda e não usa, as vezes leva a rede, dá os pescadores irem pescar com a rede, já a rede é mais louvável e devemos cuidar mais da rede porque a rede nos evita muita coisa. Disseram que demos lavar depois de três meses para tirar as poeiras** |
|  | **não me disseram nada** |
|  | **disseram que devíamos estender a rede no sol para que aquele remédio evapore e não termos comichão** |
|  | **Os que distribuíram as redes na comunidade disseram que as redes devemos cuidar bem delas para mesmo quando recebemos a visita, também podemos dar a rede para não serem picados com mosquitos não é só para mim. Pode não apanhar outras coisas mas a rede deve ter. também disseram-nos que não podemos usar antes de lavar pois os que usaram antes de lavar, acordaram com alergias pois eles foram ditos e não seguiram mas dia seguinte muitas pessoas lavaram as redes e ninguém mais teve alergia.** |
| **Em relação as mensagens sobre o uso da rede mosquiteira veiculadas pelos profissionais de saúde durante a distribuição massiva da rede mosquiteira, mais que a metade das mulheres grávidas residentes na zona rural referiram que foram ditas para lavarem as redes com OMO e secarem no sol para depois usarem, como evidenciam os depoimentos abaixo:**  *“Só nos disseram que se você estiver muito apressado, pode lavar a rede mosquiteira com bingo e estender e também pode estender no sol durante três dias”*  *“disseram que devemos lavar com OMO e sabão bingo antes de usarmos para evitar que o remédio que está na rede nos cause problemas e que também podíamos estender no sol”*  *“disseram que devemos lavar com OMO antes de usarmos para evitar que o remédio que está na rede nos cause borbulhas e outras doenças e que também para estender no calor”*  **Uma mulher grávida afirmou que durante a distribuição das redes mosquiteiras na comunidade não deviam usar indevidamente as redes mosquiteiras, como vem no depoimento abaixo:**  *“disseram que não querem ver as redes esticadas para proteger as hortas, faz de conta estás a plantar hortas e também não querem ver usarmos as redes levarmos para pescar no massacatine [lagoa] e não disseram mais nada”*  **Algumas mulheres, em relação as mensagens chave que deviam ser dadas pelos profissionais de saúde tanto na distribuição massiva assim como na CPN, deviam explicar tudo relacionado ao tratamento da rede antes de se usar para evitar que as pessoas sofram efeitos que lhes façam desistir de usar a rede.**  *“mesmo agora nos deram a rede mas não me explicara o que devo fazer antes de começar a usar por isso o ministério da Saúde devia rever isso e dizer as enfermeiras assim como os distribuidores das redes mosquiteiras na comunidade para darem uma boa explicação do uso das redes mosquiteiras. As outras abrem e usam logo e sofrem de alergia e dessa forma podem não usar mais a rede mosquiteira”*  **A maioria das mulheres gravidas referiu que durante a distribuição das redes mosquiteiras na comunidade, os distribuidores referiram que deviam lavar as redes com OMO e estenderem ao sol para que o insecticida evaporasse e não lhes criasse problemas como alergias nem sufoco:**  *“...disseram que devíamos estender a rede no sol para que aquele remédio evapore e não termos comichão.”*  *“Pode não apanhar outras coisas mas a rede deve ter. também disseram-nos que não podemos usar antes de lavar pois os que usaram antes de lavar, acordaram com alergias pois eles foram ditos e não seguiram mas dia seguinte muitas pessoas lavaram as redes e ninguém mais teve alergia.”*  *“os que distribuíram as redes na comunidade disseram que não era para lavar mas sim era para esticar no calor para que o remédio não me possa causar mal”*  *“Os distribuidores disseram que antes de eu usar a rede mosquiteira devo lavar a rede, deixar apanhar sol para desaparecer o remédio que tinha. Com muito calor, devia deixar a rede, apanhar um bom sol durante um período de dois dias ainda a estender depois de lavar. Disseram me para lavar na bacia, usando OMO e colocar na outra bacia.”*  **Algumas mulheres gravidas entrevistadas, afirmaram que durante a distribuição das redes mosquiteiras nas comunidades, foram informadas que deviam usar a rede mosquiteira para se protegerem da malária e não para usarem nos canteiros nem para protegerem fruteiras:**  *“…do mesmo jeito que falei aqui, disseram que depois de estender usar e me disseram para não usar nas saladas e se fizesse isso ia ser picada com os mosquitos”*    *“…disseram que as redes é para usarmos para prevenir dos mosquitos de modo a não termos malária não era para usarmos para cobrir os canteiros, nem mangas e nada mais”* | |

**Recomendações:** mesmo agora nos deram a rede mas não me explicara o que devo fazer antes de começar a usar por isso o ministério da Saúde devia rever isso e dizer as enfermeiras assim como os distribuidores das redes mosquiteiras na comunidade para darem uma boa explicação do uso das redes mosquiteiras. As outras abrem e usam logo e sofrem de alergia e dessa forma podem não usar mais a rede mosquiteira

| **Tema** | **Código** | **Definição do código** |
| --- | --- | --- |
| **Percepções das mulheres gravidas em relação uso adequado da REMILD para prevenção da malária** | Opinião sobre o uso da rede mosquiteira | Este código foi usado quando as entrevistadas mencionavam:   - A importância do uso da RTI’s para protegerem-se da malária - O que as entrevistadas faziam com as redes mosquiteiras, tanto para as que mencionavam estarem a usar adequadamente assim como inadequadamente - O que os vizinhos e conhecidos faziam com as redes mosquiteiras, tanto adequadamente assim como inadequadamente |
|  | Conhecimento das mães em relação aos grupos que necessitam de mais atenção na prevenção da malária (grupos vulneráveis) | Este código imergia quando:   - As entrevistadas mencionavam os grupos tradicionalmente considerados vulneráveis para malária - As entrevistadas mencionavam as razões de não serem considerados simplesmente as mulheres grávidas e crianças menores de cinco anos como grupos prioritários para o uso das RTI’s |
| **Mitos, tabus e crenças relacionados ao uso da rede mosquiteira** | Crenças relacionadas ao uso da rede mosquiteira | Este código mencionava o seguinte:   - O que as mulheres acreditavam sobre o uso da RTI’s - Opinião das mulheres e dos líderes comunitários sobre o uso da RTI |
|  | Tabús na comunidade sobre o uso da RTI’s | O código é definido pelas opiniões e crenças das mulheres sobre os boatos relacionados ao uso da RTI (Ex: as RTI’s provocam mais mosquitos, muitos insectos, etc) |
| **Barreiras comportamentais para o uso da rede mosquiteira tratada com insecticida de longa duração** | Barreiras pessoais sobre o uso das RTI’s | Este código foi usado quando as entrevistadas mencionavam as dificuldades que elas tinham para usarem adequadamente as RTI’s |
|  | Barreiras de vizinhos, familiares e outros conhecidos sobre o uso das RTI’s | Criou-se este código porque as entrevistadas não se sentiam a vontade em responder/ falar das dificuldades que tinham para usarem a RTI mas reportavam que outras pessoas mencionavam muitos problemas como alergias quando as usavam, aumento da temperatura, dificuldades em movimentar-se dentro da RTI, etc. |
|  | Preferência do uso da RTI’s por estações do ano | Este código foi usado quando as mulheres mencionavam:   - Preferência do uso das RTI’s no inverno - Preferência do uso da RTI’s no verão - Preferência do uso das RTI’s tanto no verão assim como no inverno - Motivos de preferências das estações do ano para o uso da RTI’s |
| **Mensagens chave veiculadas pelos profissionais de Saúde durante a distribuição das REMILD** | Mensagens-Chave sobre as RTI’s na distribuição massiva | O código usou-se quando as mulheres mencionavam:   - Informação recebida sobre a importância do uso da RTI - Cuidados a ter com a RTI antes do uso - Não ter recebido informação sobre o uso da RTI pelos distribuidores durante as campanhas - Ter recebido informação correcta sobre o cuidado a ter com a RTI antes do uso - Ter recebido informação incorrecta sobre o cuidado a ter com a RTI antes do uso como por exemplo: lavar a rede com detergente e estender ao sol ou estender na sombra em menos de 24 horas |
|  | Mensagens-Chave sobre as RTI’s na CPN | O código mencionava:   - Não ter recebido informação sobre o uso da RTI pelos profissionais de saúde no âmbito da entrega da RTI na CPN - Ter recebido informação correcta pelo profissional de saúde na CPN sobre o cuidado a ter com a RTI antes do uso   Ter recebido informação incorrecta na CPN sobre o cuidado a ter com a RTI antes do uso como por exemplo: lavar a rede com detergente e estender ao sol ou estender na sombra em menos de 24 horas |
| Facilitadores para uso da rece mosquiteira |  | Mesmo agora nos deram a rede, mas não me explicara o que devo fazer antes de começar a usar por isso o ministério da Saúde devia rever isso e dizer as enfermeiras assim como os distribuidores das redes mosquiteiras na comunidade para darem uma boa explicação do uso das redes mosquiteiras. As outras abrem e usam logo e sofrem de alergia e dessa forma podem não usar mais a rede mosquiteira  As coisas que iam facilitar o coiso, usar bem a rede mosquiteira, depois de nos dar as redes, deve falar nas escolas e nos ensinar a usar.  O governo deve distribuir sempre as redes mosquiteiras porque por exemplo, elas são muito caras para o dinheiro que nós trabalhamos e pode acontecer aquilo dos outros não terem o dinheiro e morrer de malária. |
|  |  |  |
