# Supplementary material for: Factors associated with the use of long-lasting insecticidal nets in pregnant women and mothers with children under five years of age in Gaza province, Mozambique
Source: PLOS Glob Public Health. 2024 Jan 16;4(1):e0002811. doi: 10.1371/journal.pgph.0002811 (PMC10790986; doi:10.1371/journal.pgph.0002811)
Supplement: S3 Text — (DOCX) [file pgph.0002811.s004.docx]

Tema: **Percepções das mães com crianças menores de cinco anos em relação uso adequado da REMILD para prevenção da malária**

| Código | Depoimentos |
| --- | --- |
| Opinião sobre o uso da rede mosquiteira | **[silencio] serve para amarar e nos proteger da malaria. Outras coisas já não sei**  **amarar para nos cobrir da malaria. Não posso dizer outras coisas porque não é isso que as pessoas dizem quando nos dão a rede mosquiteira, outras pessoas usam para cobrir as saladas e também irem pescar com as redes, eu na minha parte não faço essas coisas** |
|  | **a rede mosquiteira serve para evitar a malária**  **só estiquei para prevenir malária e não outra coisa** |
|  | **usamos só para prevenir malária**  **ter malaria, serve para evitar aquilo alí, doenças, malária, para não afectar a criança para ter malaria, não usamos para outra coisa** |
|  | **Inicialmente, eu não quero mentir, não gosto da rede mas costumo a utilizar por causa dos meus filhos. Os outros tem feito aquilo de esticar a rede e dentro da rede semearem coisas**  **a rede mosquiteira serve para evitar a doença da malária e não só a malaria, evita muita coisa, por isso temos que nos prevenir da malaria** |
|  | **Uso a rede mosquiteira para me prevenir da malária, e não ser picado com mosquitos**  **a rede mosquiteira serve para não ser picado com mosquito e apanhar a doença de malária** |
|  | **para mim é importante sim porque nos protegemos da malária e quando também estiver grávida, ajuda a prevenir da malária e a criança não sair com doença de malaria, sendo eu picado, a criança, também sairá com malária e outras doenças** |
|  | **uso para nos cobrirmos, estico para dormir com as crianças**  **a rede serve para nos cobrirmos e não apanharmos malária** |
|  | **uso para nos cobrirmos, estico para não sermos picados com mosquitos**  **a rede serve para evitar mosquitos** |
|  | **uso para nos cobrirmos, estico para não sermos picados com mosquitos**  **a rede serve para evitar mosquitos** |
|  |  |
|  | **uso para não sermos picado com mosquitos**  **na minha opinião é que uso a rede para não ter malária** |
|  | **Na minha opinião a rede é usada para não ser picada com mosquitos e não ter malária**  **uso para não sermos picado com mosquitos** |
|  | **na minha opinião a rede serve para cuidar dos mosquitos para não ter malária**  **tenho dado a utilidade a rede mosquiteira para me prevenir da malária mas tenho visto outras pessoas levarem a rede para a pesca** |
|  | **Na minha opinião a rede serve para mosquitos. Serve para que quando tiver mosquitos, usar.**  **Uso para me proteger dos mosquitos** |
|  | **para mim a rede serve para mosquitos, e também evita outras doenças porque sem a rede os mosquitos vão te picar muito**  **uso para me prevenir De mosquitos, malaria e outras doenças.** |
|  | **Uso a rede mosquiteira para dentro quando quero dormir, dormir dentro da rede.**  **[silêncio de 10 segundos] a rede mosquiteira serve para não ser picado com mosquito e evitar a doença malária**  **Para mim é importante sim porque nos protegemos da malária e quando também estiver grávida, ajuda a prevenir da malária e a criança não sair com doença de malaria, sendo eu picado, a criança, também sairá com malária e outras doenças** |
|  | **uso para me prevenir de mosquitos, malaria**  **para mim a rede serve para evitar mosquitos para não apanhar mosquitos, e também se estiver suja lavar** |
|  | **a rede mosquiteira para mim serve para esticar e dormir dentro dela**  **Estico, depois durmo dentro da rede para evitar mosquitos** |
| **Em relação a opinião que as mães com crianças menores de cinco anos tem sobre o uso da rede mosquiteira, cerca da metade referiu que só usa a rede para prevenir-se da malária assim como protegerem as crianças para não serem picadas com os mosquitos e posteriormente terem malária, como evidenciam os depoimentos abaixo:**  *“Usamos só para prevenir malária, ter malaria, serve para evitar aquilo alí, doenças, malária, para não afectar a criança para ter malaria, não usamos para outra coisa rede mosquiteira serve para evitar a doença da malária e não só a malaria, evita muita coisa, por isso temos que nos prevenir da malaria”*  *“Uso a rede mosquiteira para me prevenir da malária, e não ser picado com mosquitos*  *a rede mosquiteira serve para não ser picado com mosquito e apanhar a doença de malária.”*  *“Para mim é importante sim porque nos protegemos da malária e quando também estiver grávida, ajuda a prevenir da malária e a criança não sair com doença de malaria, sendo eu picado, a criança, também sairá com malária e outras doenças.”*  **Questionadas sobre a sua opinião em relação a rede mosquiteira assim como de pessoas próximas a elas, uma parte das mulheres entrevistadas, referiu que apenas usa a rede mosquiteira mas não gosta e as outras pessoas próximas a elas preferem não usarem a rede, levando esta para cobrirem os canteiros assim como para a pesca, vide as declarações das mulheres abaixo:**  *“Não posso dizer outras coisas porque não é isso que as pessoas dizem quando nos dão a rede mosquiteira, outras pessoas usam para cobrir as saladas e também irem pescar com as redes, eu na minha parte não faço essas coisas.”*  *“Inicialmente, eu não quero mentir, não gosto da rede mas costumo a utilizar por causa dos meus filhos. Os outros tem feito aquilo de esticar a rede e dentro da rede semearem coisas.”*  *“Tenho dado a utilidade a rede mosquiteira para me prevenir da malária mas tenho visto outras pessoas levarem a rede para a pesca.”*  **Em relação a opinião das mulheres com crianças menores de cinco anos sobre o uso da rede mosquiteira na zona rural, por unanimidade referiram que a rede mosquiteira somente serve para a prevenção contra os mosquitos e consequentemente evitar a malária conforme evidenciam os depoimentos abaixo:**  ***“para mim a rede serve para mosquitos, e também evita outras doenças porque sem a rede os mosquitos vão te picar muito***  ***uso para me prevenir De mosquitos, malaria e outras doenças.”***  ***“uso para me prevenir de mosquitos, malaria***  ***para mim a rede serve para evitar mosquitos para não apanhar mosquitos, e também se estiver suja lavar”***  ***“a rede mosquiteira para mim serve para esticar e dormir dentro dela***  ***Estico, depois durmo dentro da rede para evitar mosquitos”*** | |
| Conhecimento das mães em relação aos grupos que necessitam de mais atenção na prevenção da malária (grupos vulneráveis) |  |
|  | **pessoas grávidas e a criança** |
|  | **Bebé pequeno, mulheres grávidas, se não usarmos a rede as crianças que estão dentro vão sair com doenças, mas nós também que somos grandes devemos usar as redes mosquiteiras se não vamos apanhar malária** |
|  | **mulheres gravidas e mulheres que tem bebé pequeno, porque as crianças podem ficar doentes, terem doença de malária.** |
|  | **Bebé pequeno, mulheres grávidas e idosos** |
|  | **serve para todos, tanto as que estão gravidas, crianças e adultos devem usar para não apanharem doença de malária** |
|  | **primeiro posso dar ou esticar a rede para as crianças e as mulheres grávidas para não apanharem a doença de malária.** |
|  | **primeiro posso dar ou esticar a rede para as crianças e nós as outras pessoas podemos usar mas a prioridade são as crianças.** |
|  | **Todos devem ser protegidos para não terem malaria mas primeiro posso dar ou esticar a rede para as crianças e depois nós os adultos.** |
|  | **Todos devem ser protegidos para não terem malaria mas primeiro posso dar ou esticar a rede para as crianças e depois nós os adultos.** |
|  | **podemos dar as crianças e mulheres grávidas** |
|  | **podemos dar as crianças e mulheres grávidas** |
|  | **podemos dar as crianças e mulheres grávidas porque se não apanhar rede a mulher grávida não estará bem e também as crianças não sabem se cobrir** |
|  | **os grupos que devem ser mais cuidados, são as mulheres que acabam de dar parto e as grávidas** |
|  | **mesmo as crianças devemos proteger mais para não serem picados com mosquitos mas todos nós devemos usar a rede mosquiteira** |
|  | **os grupos que devem ser mais cuidados, são as crianças pequenas, arranjar um sitio dormirem todas juntas para poder poupar as redes e depois é que viriam os adultos assim como as grávidas** |
|  | **podemos dar as crianças e mulheres grávidas, pois podem criar facilmente doenças de malária** |
| No que tange ao conhecimento das mães sobre os grupos que necessitam de mais atenção para o uso da rede mosquiteira como forma de prevenção da malaria, mais que a metade, apontou crianças menores e mulheres gravidas, as crianças menores por não saberem se cobrir e as mulheres gravidas por temerem que estas contraiam a malária e passem para as crianças que estão a espera.  *“Bebé pequeno, mulheres grávidas, se não usarmos a rede as crianças que estão dentro vão sair com doenças, mas nós também que somos grandes devemos usar as redes mosquiteiras se não vamos apanhar malária”*  *“Mulheres grávidas e mulheres que tem bebé pequeno, porque as crianças podem ficar doentes, terem doença de malária.”*  *“Primeiro posso dar ou esticar a rede para as crianças e as mulheres grávidas para não apanharem a doença de malária.”*  *“Podemos dar as crianças e mulheres grávidas porque se não apanhar rede a mulher grávida não estará bem e também as crianças não sabem se cobrir”*  **Em contrapartida, cerca da metade das mães com crianças menires de cincos referiu que todos os grupos deviam usar a rede mosquiteira para prevenirem-se da malária porque a malária não escolhe as faixas etárias, vide os depoimentos abaixo:**  *“Todos devem ser protegidos para não terem malaria mas primeiro posso dar ou esticar a rede para as crianças e depois nós os adultos.”*  *“Serve para todos, tanto as que estão gravidas, crianças e adultos devem usar para não apanharem doença de malária”*  Metade das mães com crianças menores de cinco anos na zona rural, referiram que os grupos mais vulneráveis a malária são as mulheres grávidas, as puérperas e as crianças menores  ***“podemos dar as crianças e mulheres grávidas, pois podem criar facilmente doenças de malária”***  ***“Todos os grupos devem ser mais cuidados mas, com foco nas crianças pequenas, arranjar um sitio dormirem todas juntas para poder poupar as redes e depois é que viriam os adultos assim como as grávidas”***  **Uma parte das** mães com crianças menores de cinco anos na zona rura referiu que todos devem usar a rede mosquiteira. | |
|  |  |
|  |  |

Tema: **Mitos, tabus e crenças relacionados ao uso da rede mosquiteira**

| **Código** | **Depoimento** |
| --- | --- |
| Crenças relacionadas ao uso da rede mosquiteira |  |
|  | **aí não sei nada sobre o que se diz na comunidade, não sei mesmo e nunca ouvi nada** |
|  | **Na comunidade nunca ouvimos informações/mitos sobre o uso das redes mosquiteiras, nunca ouvi falar estranho sobre a rede mosquiteira** |
|  | **Na comunidade nunca ouvimos informações/mitos sobre o uso das redes mosquiteiras** |
|  | **outros dizem que a rede provoca uma alergia** |
|  | **não nunca ouvi nada** |
|  | **eu não sei, nunca ouvi nada, sempre falam no geral boas coisas da rede** |
|  | **eu não sei, nunca ouvi nada** |
|  | **eu não sei, nunca ouvi nada** |
|  | **só dizem que provoca alergia e também traz gripe quando você não lava, podem falar outras coisas mas eu não sei** |
|  | **nunca ouvi nada sobre a rede mosquiteira** |
|  | **nunca ouvi nada sobre a rede mosquiteira** |
|  | **nunca ouvi nada sobre a rede mosquiteira** |
|  | **diz se não podemos levar as redes usarmos como cordas** |
|  | **não nunca ouvi nada** |
|  | **eu não sei, nunca ouvi nada** |
|  | **nunca ouvi nada sobre a rede mosquiteira, para além de saber da importância da rede mosquiteira** |
| **Em relação aos mitos da comunidade quanto ao uso da rede mosquiteira, muitas mães com crianças menores de cinco anos referiram que não tem ouvido algo na comunidade sobre a rede.**  *“Na comunidade nunca ouvimos informações/mitos sobre o uso das redes mosquiteiras, nunca ouvi falar estranho sobre a rede mosquiteira”*  *“Eu não sei, nunca ouvi nada, sempre falam no geral boas coisas da rede”*  Em relação aos mitos, tabus e crenças associadas ao uso da rede mosquiteira, as entrevistadas foram unanimes em afirmar que nunca ouviram algo sobre as redes mosquiteiras que pelo contrario, os lideres comunitários, religiosos apenas incentivam o uso da rede e dão alguns conselhos sobre o uso correcto da rede.  ***“diz se não podemos levar as redes usarmos como cordas***  ***não nunca ouvi nada”***  ***“eu não sei, nunca ouvi nada”***  ***“nunca ouvi nada sobre a rede mosquiteira, para além de saber da importância da rede mosquiteira”*** | |

Tema: **Barreiras comportamentais para o uso da rede mosquiteira tratada com insecticida de longa duração**

| **Código** | | **Depoimento** | |
| --- | --- | --- | --- |
| **Barreiras pessoais e de conhecidos** | |  | |
|  |  | **nunca aconteceu nada comigo**  **heeehhhh, não sei porque eu uso a rede mosquiteira, não sei outras pessoas [silencio]… não sei** | |
|  |  | **Nunca aconteceu nada para mim.**  **Tem aquilo de utilizar sem lavar, então outros já ouvi a dizerem que lhes faz comichão mas eu sempre recomendo a não deixarem de usar** | |
|  |  | **Não sei, não sei**  **Nunca aconteceu nada para mim. Na primeira vez que usei a rede mosquiteira me fez muita comichão na cara mas depois de algum tempo passou, não precisei de ir ao hospital e não levou muito tempo** | |
|  |  | **outras dizem que não usam porque não gostam mas outras recebem a rede e usam na machamba.**  **Quando estou dentro da rede me sinto incomodada. No tempo de verão, sinto muito calor, tipo que quando estou dentro da rede aquece muito.** | |
|  | | **para mim nunca aconteceu nada. Uhmmm.**  **Algumas deixam de usar a rede mosquiteira porque dizem a rede aquece muito e quando estão dentro da rede mosquiteira ficam sem ar mas não sabem que aquilo protege muitas coisas para que a pessoa consiga apanhar a vida. E quando a pessoa usa a rede não vai apanhar a doença de malária** | |
|  |  | **nunca me aconteceu nada, só vejo outras pessoas se coçarem e saírem borbulhas, para mim nunca aconteceu nada.**  **outras pessoas falam que quando esticam a rede mosquiteira tem alergia e saem muitas borbulhas e outros dizem que não dormem bem na rede mosquiteira, outros dizem que quando dormem na rede, fica parecendo que não dormiram bem e não apanham bem o ar.** | |
|  |  | **nunca me aconteceu nada**  **As pessoas tem dito que quando não lavam a rede mosquiteira, depois saem borbulhas portanto** | |
|  |  | **As pessoas tem dito que quando estão dentro da rede não respiram bem**  **nada, nunca me aconteceu nada porque gosto da rede mosquiteira mas algumas vezes me acontecia eu entrar dentro da rede mosquiteira não me sentir bem mas nunca deixei […], não respirava bem.** | |
|  | | **heehhhhhh nunca me disseram nada e quando converso com outras pessoas nunca dizem na minha presença e os meus vizinhos também usam a rede mosquiteira. Eu já ouvi também outras pessoas a dizerem que a rede lhes causa alergias e ficam sufocadas sem apanhar o ar mas eu tenho sempre lhes incentivado a usarem a rede mosquiteira por que se forem picadas com os mosquitos, vão apanhar malária.**  **eu uso a rede, só não gosto quando estou grávida, me fazia sentir muito calor e nunca me criou alergias** | |
|  | | **[silêncio de 6 segundos], nunca me disseram nada**  **para mim nunca aconteceu nada. Porque antes de usar lavo antes de usar** | |
|  | | **nunca tive nenhuma reacção porque já me disseram que antes de usar tenho que lavar** | |
|  | | **Outras pessoas dizem que não conseguem usar a rede mosquiteira, ficam sufocadas.**  **Nunca me aconteceu nada de estranho com a rede, sempre gostei da rede e já não me sinto bem fora da rede** | |
|  | | **só dizem que provoca alergia e também traz gripe quando você não lava, podem falar outras coisas mas eu não sei**  **quando não lavo a rede me cria borbulhas mas quando lavo não me cria borbulhas. Também quando aquece, a rede aumenta o calor mas ela é boa porque não permite a entrada de mosquitos** | |
|  |  | **as vezes outras pessoas dizem que não usam a rede mosquiteira porque saem borbulhas e as outras pessoas dizem que não apanham ar** | |
|  |  | **não sei porquê não gostam da rede mas tenho escutado outras pessoas a dizerem que não gostam mas porquê muitos não dizem para mim, eu usei a rede mesmo para as minhas crenças e alguns tem dito que lhes cria aquilo e aquilo.** | |
|  |  |  | |
| **A maioria das mães com crianças menores de cincos anos entrevistadas afirmaram já terem experimentado desafios para o uso da rede como por exemplo: i) alergia, ii) sufoco e iii) aumento da temperatura, pior no verão, como mostram os depoimentos abaixo:**  *“Na primeira vez que usei a rede mosquiteira me fez muita comichão na cara mas depois de algum tempo passou, não precisei de ir ao hospital e não levou muito tempo”*  *“Quando estou dentro da rede me sinto incomodada. No tempo de verão, sinto muito calor, tipo que quando estou dentro da rede aquece muito.”*  *“Quando não lavo a rede me cria borbulhas mas quando lavo não me cria borbulhas”*  *“Por algum tempo deixei de usar a rede mosquiteira porque a rede aquece muito e quando estou dentro da rede mosquiteira fico sem ar mas sei que aquilo protege muitas coisas para que a pessoa consiga apanhar a vida.”*  **Questionadas que sabiam de algumas pessoas próximas que tem tido dificuldades no uso da rede mosquiteira, todas as mães das crianças menores de cinco anos, reportaram que conhecem pessoas que não gostam de usar a rede mosquiteira porque quando usam saem borbulhas, aumenta calor e ficam sufocadas:**  *“As vezes outras pessoas dizem que não usam a rede mosquiteira porque saem borbulhas e as outras pessoas dizem que não apanham ar”*  *“Nunca me aconteceu nada, só vejo outras pessoas se coçarem e saírem borbulhas, para mim nunca aconteceu nada.”*  *“…outras pessoas falam que quando esticam a rede mosquiteira tem alergia e saem muitas borbulhas e outros dizem que não dormem bem na rede mosquiteira, outros dizem que quando dormem na rede, fica parecendo que não dormiram bem e não apanham bem o ar”*  **Algumas mulheres também mencionaram que pessoas próximas por causa das dificuldades que tem tido (aumentar calor no verão, comichão e não gostar) com o uso da rede mosquiteira, acabam levando a rede para usar nos canteiros assim como na pesca:**  *“Outras dizem que não usam porque não gostam mas outras recebem a rede e usam na machamba.”*  *“As pessoas tem dito que quando não lavam a rede mosquiteira, depois saem borbulhas portanto. Também quando aquece, a rede aumenta o calor, por isso acabam levar a rede para pescar peixe pequeno apesar dela é boa porque não permite a entrada de mosquitos”* | | | |
| **Preferências do uso da rede mosquiteira por estacoes do ano** | | |  |
|  |  |  | **Todo período usamos a rede mosquiteira** |
|  |  |  | **Muito muito no verão, porque tem muitos mosquitos mas como o tempo já mudou, mesmo neste inverno existem muitos mosquitos mas sempre usei mesmo sendo inverno** |
|  |  |  | **Todos os dias tem que se usar a rede mosquiteira, sempre usamos a rede mosquiteira** |
|  |  |  | **eu principalmente uso no tempo de verão porque tem muitos mosquitos. No inverno uso mas não há mosquitos e as vezes esqueço. Mas no verão não há dias que não estico rede mas no inverno há dias que acho não há mosquitos.** |
|  |  |  | **eu uso em todo tempo. É obrigatório eu usar a rede em todo tempo porque se eu não fazer isso, serei bem picado com os mosquitos e ter malária** |
|  |  |  | **hahhhhhhh, qualquer hora, a rede só tiro de onde eu durmo para lavar não tenho tempo** |
|  |  |  | **uso a rede em todo o período porque em todo sítio e tempo, tem malária** |
|  |  |  | **uso a rede em todo o período porque em todo sítio e tempo, tem malária e tem mosquitos** |
|  |  |  | **uso a rede em todo o período porque em todo sítio e tempo, tem malária e tem mosquitos e eu uso sem problemas** |
|  |  |  | **uso a rede em todo o período porque em todo sítio e tempo, tem malária e tem mosquitos** |
|  | | | **uso a rede em todo o período porque em todo sítio e tempo, tem malária e tem mosquitos** |
| **Mais que a metade das mães com crianças menores de cinco anos entrevistadas na zona urbana, referiu que usa a rede mosquiteira tanto no inverno assim como no verão apesar de no inverno não sentirem mosquitos, vide as declarações abaixo:**  *“Muito muito no verão, porque tem muitos mosquitos mas como o tempo já mudou, mesmo neste inverno existem muitos mosquitos mas sempre usei mesmo sendo inverno”*  *“Todos os dias tem que se usar a rede mosquiteira, sempre usamos a rede mosquiteira”*  *“eu uso em todo tempo. É obrigatório eu usar a rede em todo tempo porque se eu não fazer isso, serei bem picado com os mosquitos e ter malária”*  *“uso a rede em todo o período porque em todo sítio e tempo, tem malária e tem mosquitos e eu uso sem problemas”*  **Algumas mães referiram que usam a rede mosquiteira no verão e no inverno não tem usado com frequência porque não há mosquitos:**  *“Eu principalmente uso no tempo de verão porque tem muitos mosquitos. No inverno uso mas não há mosquitos e muitas vezes esqueço. Mas no verão não há dias que não estico rede mas no inverno há dias que acho não há mosquitos”*  *“Uso a rede no tempo de calor porque tem muitos mosquitos para me provocarem o coiso, coiso, malária”*  **A maioria das mulheres referiu que nunca teve nenhuma barreira para o uso da rede mosquiteira e que gostavam de usar a rede assim como já não se sentiam a vontade dormindo fora dela pois antes de usarem, primeiro lavaram, vide os depoimentos abaixo:**  *“Nunca me aconteceu nada de estranho com a rede, sempre gostei da rede e já não me sinto bem fora da rede”*  *“nunca tive nenhuma reacção porque já me disseram que antes de usar tenho que lavar”*  *“para mim nunca aconteceu nada. Porque antes de usar lavo antes de usar”*  **As mesmas mulheres questionadas sobre o que fazia com que os seus vizinhos e conhecidos não usassem a rede mosquiteira, foram unânimes em afirmar que estas não usam por se sentirem sufocadas dentro da rede assim como provocar-lhes alergia, conforme mostram os depoimentos abaixo:**  *“heehhhhhh nunca me disseram nada e quando converso com outras pessoas nunca dizem na minha presença e os meus vizinhos também usam a rede mosquiteira. Eu já ouvi também outras pessoas a dizerem que a rede lhes causa alergias e ficam sufocadas sem apanhar o ar mas eu tenho sempre lhes incentivado a usarem a rede mosquiteira por que se forem picadas com os mosquitos, vão apanhar malária.”*  *“Outras pessoas dizem que não conseguem usar a rede mosquiteira, ficam sufocadas.”*  **Uma mulher referiu que gosta de usar a rede mosquiteira mas quando estiver grávida não gosta de usar pois sufoca-lhe, causa calor e alergia. Vide o depoimento abaixo:**  *“eu uso a rede, só não gosto quando estou grávida, me fazia sentir muito calor e nunca me criou alergias”*  **Mais que a metade das mães com crianças menores de cinco anos entrevistadas, referiram que a rede mosquiteira elas usam tanto no inverno assim como no verão, dado que em todas as estacoes de ano existem os mosquitos, conforme os depoimentos abaixo:**  *“tanto no inverno assim como no verão uso a rede mosquiteira pois em todo momento existem os mosquitos”*  *“eu uso em todo tempo. É obrigatório eu usar a rede em todo tempo porque se eu não fazer isso, serei bem picado com os mosquitos e ter malária”*  *“uso a rede em todo o período porque em todo sítio e tempo, tem malária e tem mosquitos em todo o tempo”*  **Algumas mulheres, relataram que usam mais a rede mosquiteira no verao pois, é nesta altura do ano que verifica-se maior população dos mosquitos do que no inverno que não há necessidade do seu uso. Vide o depoimento abaixo:**  *“mesmo agora no inverno usamos mas não é igual ao verão, pois há muitos mosquitos. Outros dias agora no inverno não uso por esquecer devido a falta de necessidade do uso desta”* | | | |
|  |  | | |
| **Experiencia do uso da rede mosquiteira no primeiro dia** |  | | |
|  | **meti na agua, estendi no estendal por 30 minutos, na sombra e usei sem nenhum problema.** | | |
|  | **não me aconteceu nada, só usei bem e sem problemas** | | |
|  | **Lavei a rede mosquiteira com omo e estendi na sombra por um período de 30 minutos** | | |
|  | **não foi difícil, isso não sei se é que desde na minha crescença, sempre dormi na rede mosquiteira.** | | |
|  | **só estendi, não lavei, só estendi na esteira e usar** | | |
|  | **quando me deram a rede, disseram que devíamos colocar no calor para sair o remédio que está na rede e depois eu coloquei no sol durante dois dias e depois levei para usar, pus na água, lavei com OMO e depois estendi** | | |
|  | **só levei a rede mosquiteira, lavei, estendi e depois levei para usar** | | |
|  | **só levei a rede mosquiteira, lavei, estendi e depois levei para usar** | | |
|  | **só levei a rede mosquiteira, lavei, estendi e depois levei para usar em seis lugares** | | |
|  | **só levei a rede mosquiteira, estendi para reduzir o remédio que tem na rede** | | |
|  | **só levei com bingo a rede mosquiteira, estendi na sombra e no anoitecer do mesmo dia me cobri, não senti nada estranho** | | |
|  | **não me aconteceu nada, apenas lavei com OMO e estendi no calor para secar e depois usamos** | | |
|  | **só lavei com água, estendi, só estendi na esteira e usar** | | |
|  | **não lavei a rede, só estiquei em casa pronta para usar e também não estendi para arejar.** | | |
|  | **só lavei e, estendi e usei a rede mosquiteira** | | |
|  | **não me aconteceu nada, apenas lavei com OMO e estendi no calor para secar e depois usamos** | | |
| **Algumas mulheres entrevistadas referiram que na primeira vez que receberam a rede mosquiteira, lavaram com bingo ou água, estenderam na sombra e depois usaram para se protegerem da malária.**  ***“Meti na água, estendi no estendal por 30 minutos, na sombra e usei sem nenhum problema.”***  ***“Só levei com bingo a rede mosquiteira, estendi na sombra e no anoitecer do mesmo dia me cobri, não senti nada estranho”***  **Algumas mães de crianças menores de cinco anos, referiram que a primeira vez que receberam a rede, lavaram com OMO e estenderam no sol/calor**  ***“Quando me deram a rede, disseram que devíamos colocar no calor para sair o remédio que está na rede e depois eu coloquei no sol durante dois dias e depois levei para usar, pus na água, lavei com OMO e depois estendi”***  ***“Lavei a rede mosquiteira com OMO e estendi na sombra por um período de 30 minutos”***  **Algumas mulheres afirmaram que na primeira experiência que tiveram com a rede mosquiteira, só tiraram do plástico e estenderam na cama ou na esteira para o uso, conforme os depoimentos abaixo:**  ***“só estendi, não lavei, só estendi na esteira e usar”***  ***“não me aconteceu nada, só usei bem e sem problemas, estendi na cama só”***  **Em relação a experiencia do uso da rede mosquiteira no primeiro dia, quase todas as mulheres referiram que só lavaram a rede mosquiteira com OMO e esticaram para prevenirem-se da malária:**  *“só lavei com OMO, estendi e usei a rede mosquiteira”*  *“só lavei com água e OMO, estendi, só estendi na esteira e usar”*  *“não me aconteceu nada, apenas lavei com OMO e estendi no calor para secar e depois usamos”*  **Uma mulher referiu que não lavou a rede mosquiteira, apenas estendeu para arejar e depois usou na cama:**  *“não lavei a rede, só estiquei em casa pronta para usar e também não estendi para arejar.”* | | | |

**Tema: Mensagens chave veiculadas pelos profissionais de Saúde durante a distribuição das REMILD**

| **Código** | **Depoimento** | | |
| --- | --- | --- | --- |
| **Mensagens sobre o uso da rede mosquiteira veiculadas pelos profissionais de saúde durante a distribuição da rede na Consulta Pré-Natal** |  | | |
|  | **disseram que é para me manter dentro da rede e não me disseram mais nada** | | |
|  | **Não disseram nada só nos deram as redes mosquiteiras e não explicaram** | | |
|  | **disseram para fazer aquilo alí, psiu, aquilo ali, esticar a rede todos os dias, não dissera mais nada… choro de criança** | | |
|  | **disseram que tenho que tenho que estar sempre dentro da rede e não me falaram nada. Tenho escutado com pessoas de fora que antes de usar a rede devemos usar a rede.** | | |
|  | **as enfermeiras disseram que devia esticar a rede lá dentro de casa para me proteger de mosquitos** | | |
|  | **disseram que está aqui a rede mosquiteira é para tirar do plástico estender no sol para poder sair o remédio que tem e depois usar** | | |
|  | **aqui no hospital não nos disseram nada, só nos deram a rede, isso fomos ditos com aqueles que distribuíram a rede nas comunidades.** | | |
|  | **aqui no hospital só disseram para usar a rede mosquiteira para nos proteger da malária, não disseram mais nada** | | |
|  | **aqui no hospital disseram quando me deram a rede que devo lavar a rede e esticar para não ser picado com mosquitos. Disseram que devia lavar com bingo e estender na sombra mas já não me lembro o tempo que devemos deixar a rede estendida.** | | |
|  | **me disseram para estender na sombra para reduzir o remédio, não me disseram para lavar.** | | |
|  | **Aqui no hospital disseram que devo usar a rede mosquiteira, esticar onde eu durmo para não ter malária** | | |
|  | **aqui só me disseram que não é para levar a rede usar como corda, é sim para me prevenir dos mosquitos para não apanhar doenças. E não me falaram sobre os cuidados a ter com a rede mosquiteira** | | |
|  | **Só dissera para usar para nos prevenir da malaria** | | |
|  | **as enfermeiras disseram que devia lavar antes de usar para não termos alergia** | | |
|  | **Aqui só me disseram que devia usar a rede mosquiteira para me prevenir da malária se não este que está aqui dentro da barriga pode sair com malária ou com outras doenças e não disseram mais nada.** | | |
|  | **Aqui no hospital só me deram a rede mosquiteira e não disseram mais nada** | | |
| **A maioria das mães com crianças menores de cinco anos, referiu que durante a distribuição da rede nas consultas pré-natais, apenas foram ditos para usarem a rede mosquiteira como forma de prevenirem-se dos mosquitos e consequentemente da malária, nada foram ditas sobre os cuidados a ter com esta antes do seu uso, como evidenciam os depoimentos abaixo.**  ***“Disseram que é para me manter dentro da rede e não me disseram mais nada. Não disseram nada só nos deram as redes mosquiteiras e não explicaram”***  ***“Disseram para fazer aquilo alí, psiu, aquilo ali, esticar a rede todos os dias, não dissera mais nada… choro de criança”***  ***“Disseram que tenho que tenho que estar sempre dentro da rede e não me falaram nada. Tenho escutado com pessoas de fora que antes de usar a rede devemos usar a rede.”***  ***“As enfermeiras disseram que devia esticar a rede lá dentro de casa para me proteger de mosquitos”***  **Algumas mães, referiram que durante a distribuição da rede na CPN, foram ditas para antes de usarem, deviam estender ao sol por algum período.**  *“aqui no hospital não nos disseram nada, só nos deram a rede, isso fomos ditos com aqueles que distribuíram a rede nas comunidades.”*  *“disseram que está aqui a rede mosquiteira é para tirar do plástico estender no sol para poder sair o remédio que tem e depois usar”*  **Algumas mães, referiram que durante a distribuição da rede na CPN, foram ditas para que antes de usarem, deviam lavar com bingo e estenderem na sombra como forma de evitar com que o insecticida lhes provoque alergias e sufoco.**  *“Me disseram para estender na sombra para reduzir o remédio, não me disseram para lavar.*  *aqui no hospital disseram quando me deram a rede que devo lavar a rede e esticar para não ser picado com mosquitos.”*  *“Disseram que devia lavar com bingo e estender na sombra mas já não me lembro o tempo que devemos deixar a rede estendida.”*  **Quase todas as mães com crianças menores de cincos anos referiram que durante a recepção da rede mosquiteira na consulta pré-natal, foram informadas pelas enfermeiras que devia usar a rede mosquiteira para protegerem-se da malária assim como protegerem as crianças que tinham:**  ***“Aqui só me disseram que não é para levar a rede usar como corda, é sim para me prevenir dos mosquitos para não apanhar doenças. E não me falaram sobre os cuidados a ter com a rede mosquiteira”* (Mãe com criança menor de cinco anos, zona rural)**  ***“Só dissera para usar para nos prevenir da malaria”* (Mãe com criança menor de cinco anos, zona rural)**  ***“Aqui só me disseram que devia usar a rede mosquiteira para me prevenir da malária se não este que está aqui dentro da barriga pode sair com malária ou com outras doenças e não disseram mais nada.”* (Mãe com criança menor de cinco anos, zona rural)**  *“Aqui no hospital só me deram a rede mosquiteira e não disseram mais nada”* (Mãe com criança menor de cinco anos, zona rural)  **Uma das mães com criança menor de cinco anos entrevistada referiu que no âmbito da oferta da rede mosquiteira na consulta pré-natal a enfermeira informou que antes de usar a rede mosquiteira, ela devia lavar a mesma para evitar alergias:**  *“As enfermeiras disseram que devia lavar antes de usar para não termos alergia”* (Mãe com criança menor de cinco anos, zona rural) | | | |
| **Mensagens sobre o uso da rede mosquiteira veiculadas pelos profissionais de saúde durante a distribuição massiva da rede mosquiteira** | |  | |
|  |  | **disseram que é para me manter dentro da rede e não me disseram mais nada** | |
|  |  | **disseram para estender no sol, outras coisas já esqueci mas não era para tirar logo.** | |
|  |  | **Não disseram nada só nos deram as redes mosquiteiras e não explicaram** | |
|  |  | **as pessoas que entraram na minha casa não chegaram de dar dicas, só entraram na minha casa, perguntaram quantos eramos e depois deram redes, não deram dicas.** | |
|  |  | **só me disseram que devia usar a rede mosquiteira e que quando sujar, lavar e estender na sombra.** | |
|  |  | **disseram só para usar as redes mosquiteiras e que não era para deixar de usar, também devíamos cobrir as crianças e não disseram mais nada** | |
|  |  | **disseram que devíamos estender na sombra, o tempo/duração já não sei. E que também não devíamos estender a rede no sol e já não me recordo de outras coisas** | |
|  |  | **Esses de distribuírem as redes em casa, não disseram nada, só nos deram a rede** | |
|  |  | **Quando distribuíram a rede eu não estava por isso não sei o que disseram.** | |
|  |  | **Quando distribuíram a rede eu não estava por isso não sei o que disseram.** | |
|  |  | **disseram que estas redes é para tirarem para arejar se não fazermos isso, o veneno que a rede tem para matar mosquitos, pode nos causar problemas.** | |
|  | | **só me disseram que devia usar a rede mosquiteira para nos proteger dos mosquitos** | |
|  | | **disseram para lavar com OMO, estender no calor para secar e depois esticar dentro de casa.** | |
|  | | **disseram que devemos usar a rede mosquiteira quando formos a dormir assim como antes de dormir quando estivermos a conversar lá fora pois as vezes tem tido muitos mosquitos, depois de esticar fora, levar para dentro de casa e não disseram nada sobre lavar e estender antes de usar.** | |
|  | |  | |
|  | |  | |
|  | |  | |
| **Mais que a metade das mães com crianças menores de cinco anos residentes na zona urbana referiu que durante as campanhas de distribuição massiva das rede mosquiteiras apenas foram ditas para usarem a rede mosquiteira e como deviam usar não foram informadas, vide os depoimentos abaixo:**  *“Disseram que é para me manter dentro da rede e não me disseram mais nada”*  *“Não disseram nada só nos deram as redes mosquiteiras e não explicaram”*  *“As pessoas que entraram na minha casa não chegaram de dar dicas, só entraram na minha casa, perguntaram quantos eramos e depois deram redes, não deram dicas.”*  *“Esses de distribuírem as redes em casa, não disseram nada, só nos deram a rede”*  **Algumas mulheres com crianças menores de cinco anos residentes na zona urbana referiram que durante a distribuição das redes mosquiteiras foram informadas para que antes de usarem a rede mosquiteira estendessem na sombra para arejar por um período que já não se lembravam para evitar com que o insecticida lhes criasse problemas, como ilustram os depoimentos abaixo:**  *“disseram que estas redes é para tirarem para arejar se não fazermos isso, o veneno que a rede tem para matar mosquitos, pode nos causar problemas.”*  *“disseram que devíamos estender na sombra, o tempo/duração já não sei. E que também não devíamos estender a rede no sol e já não me recordo de outras coisas”*  **Uma mãe entrevistada referiu que na distribuição massiva das redes mosquiteiras, foi dita para antes do uso rede estender no sol por algum período não especificado:**  *“Disseram para estender no sol, outras coisas já esqueci mas não era para tirar logo.”*  **A maioria das mães com crenças menores de cinco referiram terem sido informadas pelos distribuidores das redes mosquiteiras sobre a importância do uso da rede mosquiteira e como deveriam usa-la fora e dentro de casa porém, nada foram informadas sobre deixar arejar a rede ou lavar para reduzir o efeito adverso do insecticida impregnado na rede mosquiteira, vide os depoimentos abaixo:**  ***“Disseram que devemos usar a rede mosquiteira quando formos a dormir assim como antes de dormir quando estivermos a conversar lá fora pois as vezes tem tido muitos mosquitos, depois de esticar fora, levar para dentro de casa e não disseram nada sobre lavar e estender antes de usar.”* (Mãe com criança menor de cinco anos, zona rural)**  **“*Só me disseram que devia usar a rede mosquiteira para nos proteger dos mosquitos”* (Mãe com criança menor de cinco anos, zona rural)**  **Algumas mães com crianças menores de cinco anos na zona rural referiram que durante a distribuição da rede mosquiteira, os distribuidores referiram que estas deviam usar detergente em pó para lavar a rede e estenderem ao sol antes do uso desta, como ilustra o depoimento abaixo:**  **“disseram para lavar com OMO, estender no calor para secar e depois esticar dentro de casa.” (Mãe com criança menor de cinco anos, zona rural)** | | | |
| **Facilitadores para o uso da rede mosquiteira** | | | Para mim e minha família, o que é importante, deve a  Saúde continuar a distribuir as redes mosquiteiras”  A saúde também devia colocar as escolas, os nossos  chefes da localidade para poderem nos fazer aprender a usar a rede mosquiteira e também nos fazerem  policiamento para não usar no coiso, nas mangas e na  pesca. |
