# Supplementary material for: Factors associated with the use of long-lasting insecticidal nets in pregnant women and mothers with children under five years of age in Gaza province, Mozambique
Source: PLOS Glob Public Health. 2024 Jan 16;4(1):e0002811. doi: 10.1371/journal.pgph.0002811 (PMC10790986; doi:10.1371/journal.pgph.0002811)
Supplement: Attachment — Submitted filename: PGPH-D-23-01427.pdf [file pgph.0002811.s005.pdf]

PLOS Global Public Health

Factors associated with the use of mosquito nets in pregnant women and mothers with children under five years of age in Gaza province, Mozambique

--Manuscript Draft--

Manuscript Number:	PGPH-D-23-01427
Article Type:	Research Article
Full Title:	Factors associated with the use of mosquito nets in pregnant women and mothers with children under five years of age in Gaza province, Mozambique
Short Title:	Factors associated with mosquito net use in Mozambique
Corresponding Author:	Isabelle Munyangaju, M.D, MSc. ID, MSc. Vaccinology, MBA<br>ISGLOBAL: Instituto de Salud Global de Barcelona<br>Barcelona, Barcelona SPAIN
Order of Authors:	Isabelle Munyangaju, MD
	Amancio Vicente Nhangave, BSc
	Dulce Ósorio, MD
	Edy Nacarapa, MD
	Sozinho Ndima, MSc
	Alfa Moiane, BSc
	Ismail Chiposse, BSc
	Izaidino Muchanga, MSc
	Abuchahama Saifodine, MD
Keywords:	malaria, insecticide, prevention, infectious diseases
Abstract:	Introduction<br><br>Malaria remains a major public health concern worldwide. Malaria is endemic in Mozambique, with seasonal fluctuations throughout the country. Although the number of malaria cases in Mozambique have dropped by 11% from 2020 to 2021, there are still hotspots in the country with persistent high incidence and low insecticide-treated bed net usage.<br><br>Objective<br><br>The aim of this study is to evaluate the behavioural factors associated with the use of mosquito nets by pregnant women and women with children under 5 years old in two hotspot districts in the Gaza province.<br><br>Methods<br><br>A descriptive, qualitative cross-sectional study was conducted between June 15th and 21st 2022. An in-depth interview process was conducted with pregnant women and mothers with children under five years old, exploring their beliefs, experiences, and perception of messages conveyed by health professionals when mosquito nets were being supplied.<br><br>Findings<br><br>A total of 48 women participated (24 pregnant women and 24 women with children under 5 years). Most participants recognized the protective effects of mosquito nets in preventing malaria, and understood that women and children were high risk groups. Participants reported side effects such as allergies associated with the use of the net. They showed differing knowledge on the correct handling and usage of the mosquito net.

Powered by Editorial Manager® and ProduXion Manager® from Aries Systems Corporation

	Conclusion<br><br>There is a knowledge gap amongst the participants and their communities on the correct use of the mosquito nets. Monitoring coverage of insecticide-treated bed net use should be accompanied by regular community awareness campaigns, regular monitoring of correct use and extensive community engagement.
**Additional Information:**	
**Question**	**Response**
**Financial Disclosure**<br><br>Enter a financial disclosure statement that describes the sources of funding for the work included in this submission and the role the funder(s) played. This includes grants and any commercial funding of the work or authors.<br><br>This statement will be typeset if the manuscript is accepted for publication.<br><br>*Please review the submission guidelines and the instructions link below for detailed requirements and guidance.*	The author(s) received no specific funding for this work.
**Competing Interests**<br><br>On behalf of all authors, disclose any competing interests that could be perceived to bias this work.<br><br>This statement will be typeset if the manuscript is accepted for publication.<br><br>*Please review the instructions link below and PLOS Global Public Health's competing interests policy to determine what information must be disclosed at submission.*	The authors have no competing interest to declare.
**Data Availability**<br><br>Before publication, Authors are required to make fully available and without	The datasets analysed during the current study are available from the corresponding author on reasonable request.

restriction all data underlying their findings. Please see our PLOS Data Policy page for detailed information on this policy.<br><br>A **Data Availability Statement**, detailing where the data can be accessed, is required at first submission. Insert your Data Availability Statement in the box below.<br><br>Please see the data reporting section of our submission guidelines for instructions on what you need to include in your Data Availability Statement.<br><br>This statement will be typeset if the manuscript is accepted for publication.<br><br>*PLOS allows rare exemptions to address legal and ethical concerns. If you have legal or ethical restrictions, please use the box below to detail these in full sentences for the Journal team to consider.*	

Manuscript

Click here to access/download;Manuscript;Manuscript_final.docx

Factors associated with the use of mosquito nets in pregnant women and mothers with children under five years of age in Gaza province, Mozambique

Amancio Vicente Nhangave[1,6], Isabelle Munyangaju[2#], Dulce Ósorio[2], Edy Nacarapa[2], Sozinho Ndima[3], Alfa Moiane[4], Ismail Chiposse[1], Izaidino Muchanga[5], Abuchahama Saifodine [4, 6]

Affiliations:

1. Gaza Provincial Research Nucleus, Provincial Health Directorate, Xai-Xai, Mozambique
2. Tinpswalo Association, Vincentian Association to Fight AIDS and TB, Mozambique
3. Department of Community Health, Faculty of Medicine, Eduardo Mondlane University, Mozambique
4. National Malaria Control Programme, Gaza Provincial Health Directorate, Xai-Xai, Mozambique
5. Faculty of Health Science, University of Saint Thomas, Gaza Campus, Mozambique
6. Faculty of Medicine, Eduardo Mondlane University, Mozambique

# corresponding author: imunyangaju@gmail.com

Abstract

Introduction

Malaria remains a major public health concern worldwide. Malaria is endemic in Mozambique, with seasonal fluctuations throughout the country. Although the number of malaria cases in Mozambique have dropped by 11% from 2020 to 2021, there are still hotspots in the country with persistent high incidence and low insecticide-treated bed net usage.

Objective

The aim of this study is to evaluate the factors associated with the use of mosquito nets by pregnant women and women with children under 5 years old in two hotspot districts in the Gaza province.

Methods

A descriptive, qualitative cross-sectional study was conducted between June 15th and 21st 2022. An in-depth interview process was conducted with pregnant women and mothers with children under five years old, exploring their beliefs, experiences, and perception of messages conveyed by health professionals when mosquito nets were being supplied.

Findings

A total of 48 women participated (24 pregnant women and 24 women with children under 5 years). Most participants recognized the protective effects of mosquito nets in preventing malaria, and understood that women and children were high risk groups. Participants reported side effects such as allergies associated with the use of the net. They showed differing knowledge on the correct handling and usage of the mosquito net.

Conclusion

There is a knowledge gap amongst the participants and their communities on the correct use of the mosquito nets. Monitoring coverage of insecticide-treated bed net use should be accompanied by regular community awareness campaigns, regular monitoring of correct use and extensive community engagement.

Introduction

Malaria remains a major public health concern worldwide. According to the World Health Organization (WHO), an estimated 247 million cases of malaria and 619,000 deaths occurred worldwide in 2021. There were 95% of cases and deaths in the WHO African region alone, and 55% of all cases and 50% of deaths occurred in 6 countries in the region, including Mozambique (WHO, 2022).

Malaria is endemic in Mozambique, with seasonal fluctuations throughout the country. There is a wide variation in malaria prevalence across provinces, with higher prevalence rates in northern provinces (e.g. 57.3% in Cabo Delgado) than southern provinces (e.g. 1% in Maputo city, 16.9% in Gaza). There was also a high prevalence of malaria in rural areas and among children in Mozambique. Children under 5 years of age and pregnant women are considered high-risk groups (1). The number of malaria cases in Mozambique dropped by 11% from 11.3 million in 2020 to 10.6 million in 2021 following targeted campaigns (2).

On a three-year basis, Mozambique distributes massive amounts of insecticide-treated nets (ITNs) across all district, as part of its malaria prevention strategies, but the country still ranks fourth among the 11 countries with the highest malaria burden in estimated malaria cases and deaths(3).

According to the Malaria Control Strategic Plan 2017-2022, 85% of the population should be covered with at least one vector control intervention (indoor residual spraying and use of ITNs) in all districts(4). According to the Malaria Indicators Survey (IIM) conducted in Mozambique in 2018, 18% of respondents reported not sleeping under a mosquito net the night before the survey. The majority had no mosquito net at home, while 15% said they didn't use the net because there weren’t any mosquitoes and 10% didn't like it(5).

It was found in IIM 2018 that Gaza province had the second lowest mosquito net usage percentage, around 66.8%, with at least one mosquito net for two people at the time of the survey, while Cabo Delgado province had the highest rate of 88.8%. Among children under 5, Gaza had a lower percentage of children who slept under mosquito nets before the survey, with 66.3%, compared with Cabo Delgado province, with 92.9% (5).

Approximately a third of Gaza's malaria cases come from Chibuto and Limpopo districts. Despite the widespread distribution of ITNs every three years in these Gaza districts, malaria cases remain alarming despite the implementation of this initiative (6). Several studies have been conducted to understand factors that influence mosquito net usage to reduce malaria burden, but few have addressed the quality of key messages provided by net distributors in communities and health facilities (mass distributions of mosquito nets and antenatal consultations).

The aim of this study is to evaluate the factors associated with the use of mosquito nets by pregnant women and women with children under 5 years old in Limpopo and Chibuto districts.

Materials and Methods

Study design

A descriptive, qualitative cross-sectional study was conducted between June 15th and 21st 2022. An in-depth interview process was conducted with pregnant women and mothers with

children under five years old, exploring their beliefs, experiences, and perception of messages conveyed by health professionals when mosquito nets were being supplied.

Study site and population

The study was carried out in two rural districts of Gaza Province in Mozambique - Chibuto and Limpopo. With 154,121 residents, Limpopo district has seven health units. Chibuto is home to 222,540 people and 19 health care facilities. In both districts, agriculture, cattle raising, and fishing are the primary economic activities (7).

We randomly selected one high volume and one low volume health facility in each district. In Chibuto district the following centres were selected: Centro de Saúde de Chibuto and Centro de Saúde de Chaimite; and in Limpopo district: Centro de Saúde de Chicumbane and Centro de Saúde de Licilo, high and low volume respectively.

Participant selection and sample size

Pregnant women were identified at the antenatal clinic (ANC). Mothers of children under 5 years were identified in the outpatient (OPD) consultations, in the post-natal consultations, the consultations of children at risk (CCR) and healthy children (CCS).

The study investigator explained the study and study procedures to all the women before their consultation. The clinician screened the women for eligibility to the study during the consultation (**see Table 1**), and only those who were eligible were recruited and sent to the investigator. Following that, each woman was interviewed individually in a private consultation room at the health facility by the study investigator.

Table 1: Inclusion and exclusion criteria

Group	Inclusion criteria	Exclusion criteria
Pregnant women	• Pregnant women ≥ 18 years in the ANC<br>• Given informed consent for the study	• Pregnant women with mental health disorder
Mothers of children under 5 years old	• Mothers ≥18 years old with children under 5 years old coming for consultation at the health facility<br>• Given informed consent for the study	• Women accompanying children under 5 years who are not their actual mothers

In both groups, convenient non-probabilistic sampling was used based on the availability of potential study participants and their consent. An overall sample size of 48 women was determined by data saturation; 24 in Chibuto (14 in CS Chibuto, 10 in CS Chaimite) and 24 in Limpopo (14 in CS Chicumbane, 10 in CS Licilo) (**see Table 2**).

Table 2: Distribution of the sample size per health facility

District	Health facility	Sample	
**Chibuto**	**CS Chibuto**	Pregnant women	7

		Mothers with children under 5 years	7
	**CS Chaimite**	Pregnant women	5
		Mothers with children under 5 years	5
**Limpopo**	**CS Chicumbane**	Pregnant women	7
		Mothers with children under 5 years	7
	**CS Licilo**	Pregnant women	5
		Mothers with children under 5 years	5
**Total**			**48**

Procedure

During morning health talks conducted at both health facilities, study investigators identified participants in both groups. Potential participants were informed of the study's procedures and importance. After their routine consultations, they were informed that if they wished to participate, they would be accompanied by a study team member to an in-depth interview room after giving their informed consent.

Three well-trained study surveyors conducted in-depth interviews in a separate consultation room so as to protect patient privacy. A 45-minute interview, including informed consent, was conducted for each person. An average of four interviews were conducted per day by each surveyor. We conducted the interviews in Portuguese or Changana (local languages) and recorded them. Following transcription and translation (for interviews conducted in Changana), the interviews were analysed.

All participants were interviewed using an interview guide (Annex 1). It included closed questions to characterize them and open questions to determine their interests.

Data management and analysis

After recording the in-depth interviews on paper, the transcripts were transferred to Microsoft Word (2010) with questions and responses arranged by participant placement. There were two databases created, one for pregnant women and one for women with children under five. The instruments and informed consents used in the study were kept in a secure locker at the Gaza Provincial Health Directorate, with only the principal investigator having access. The archive will be stored for 5 years before being destroyed.

The transcripts were read by three investigators. Based on the discussion of patterns of facts and events that regularly came up within and between the groups, the three investigators constructed emerging themes. A set of codes along with their respective guidelines for applying them were developed in response to these emerging themes. Each investigator coded the transcripts independently, and they held regular meetings to discuss and reconcile their respective codes to ensure agreement. Coding the transcripts and extracting coding reports were done using Maxqda software, version 12, which was then used to prepare the data

reduction and summary tables. In the final phase of data analysis, all researchers discussed the results.

Ethics statement

This study was conducted according to the Declaration of Helsinki principles and obeyed Good Clinical Practice (GCP) guidelines. Ethical approval (for protocol and written informed consent documents) was granted by the Institutional Review Board of the Faculty of Health and the Central Hospital of Maputo, the Gaza Institutional Ethics Committee and the scientific council of the Faculty of Health of the University of Eduardo Mondlane. Considering that the study involved vulnerable populations, it was also submitted and approved by the National Health Bioethics Committee (CNBS) in Mozambique (ref # 34/CNBS2022).

Results

1. *Characteristics of the participants*

Over half of the 48 women interviewed (28/48) were between 18 and 27 years old, had a primary school education, were stay-at-home women (those who reported no paid activity on a daily, weekly or monthly basis) and were married (including de facto unions). Sociodemographic characteristics are summarized in table 3.

Table 3. Socio-demographic characteristics of participants

Characteristics	Frequency (%)	
	Mothers with children under 5 years old	Pregnant women
Age		
18 – 22	12 (50.0)	4 (16.7)
23 – 27	5 (20.8)	7 (29.2)
28 – 32	2 (8.3)	6 (25.0)
33 – 37	2 (8.3)	4 (16.7)
38 – 42	2 (8.3)	2 (8.3)
≥ 43	1 (4.2)	1 (4.2)
Education level		
No formal education	3 (12.5)	4 (16.7)
Primary	14 (58.3)	11 (45.8)
Secondary	7 (29.2)	9 (37.5)
Marital status		
Single	3 (12.5)	6 (25.0)
Married/Union	20 (83.3)	18 (75.0)
Widow	1 (4.2)	0
Divorced/separated	0	0
Occupation		
Stay-at-home	22 (91.7)	21 (87.5)

Employed (incl. civil servants)	1 (4.2)	1 (4.2)
Self-employed	1 (4.2)	2 (8.3)

2. *Knowledge about the correct use of mosquito nets*

a. General perceptions on the use of mosquito nets

Pregnant women reported that the mosquito net protected them from mosquitoes and thereby prevented malaria in over half (20/24) of the cases:

"*The mosquito net is important because it helps when there are mosquitoes to protect me and my children from getting malaria. I wash the net, stretch it out in the sun*." (PW number 07, 23 years old)

"...the mosquito net is useful for many things. It kills mosquitoes and protects us from getting malaria." (PW number 16, 34 years old)

According to 17% of pregnant women (4/24), mosquito nets provide additional protection against other diseases as well:

"In my opinion, apart from protecting us from malaria, it can protect us from getting cholera and many other diseases" (PW number 02, 18 years old)

"The mosquito net protects from mosquitoes, to prevent malaria. It also serves to avoid other diseases" (PW number 21, 39 years old)

In addition, the majority (18/24) of **mothers with children under 5** believe mosquito nets prevent malaria by keeping mosquitoes at bay:

"... By using a mosquito net, you can avoid being bitten by mosquitoes and catching malaria." (M number 24, 38 years old)

"for me, the net is good for mosquitoes, and it also prevents other diseases because without the net, mosquitoes will bite you a lot" (M number 16, 22 years old)

It was reported by two mothers with children under five years of age that they used mosquito nets to prevent malaria, but some also used the nets to fish:

"...I have used the mosquito net to prevent malaria, but I have seen other people take the net for fishing" (M number 14, 42 years old)

"...I use it to protect myself from mosquitoes, and prevent malaria and other diseases. But there are other people who use the mosquito net for fishing" (M number 13, 20 years old)

b. General perceptions on the use of mosquito nets in vulnerable groups

Over 50% of **pregnant women** (13/24) believe that malaria must be prevented for children and pregnant women by using mosquito nets. In order to avoid infecting the foetus, pregnant women should stay healthy; and healthy children need protection because of their fragile immune systems:

"I can prioritise pregnant women and children. The child has the right to sleep inside the mosquito net every day, and not just the child, the adult too, because the disease does not choose. They must sleep inside the mosquito net because if I get bitten by a mosquito, as soon as I'm pregnant, I may not feel anything, but the disease can affect what's inside me, now, when the baby comes out, it comes out with diseases, many diseases, in short, I already run the risk of being in the hospital." (PW number 01, 18 years old)

In the case I get a few nets, I'll give them to pregnant women and the little ones, since adults can protect themselves with blankets, but kids don't know how to protect themselves." (PW number 12, 22 years old)

7 out of 24 pregnant women believe that all groups (pregnant women, children, adults, and elderly) are vulnerable to malaria, therefore mosquito nets should be used by all of them:

"The net is a plus and should be used by all groups, but especially for pregnant women so that they have a healthy pregnancy and their baby is not born ill" (PW number 16, 34 years old)

"We should all sleep inside the mosquito net and I don't think there is a specific group for using the mosquito net" (PW number 11, 33 years old)

According to two pregnant women, pregnant and postpartum women under 25 years of age are the most vulnerable to mosquito bites due to their limited level of responsibility for caring for their children:

"For me, the priority should be pregnant women and women with very small babies as well as the young women who become mothers before age 25. The very young mothers under 18 years or under 25 years are not responsible enough to protect themselves and their babies." (PW number 18, 23 years old)

19 out of 24 **women with children under five years** believe that women and children are the most vulnerable groups:

"we can give it to children and pregnant women because if they don't get a net, the pregnant woman won't be well and the children don't know how to cover themselves" (M number 16, 22 years old)

"Small babies, pregnant women, if we don't use the net, the children inside will come out with illnesses, but we, who are big, should use mosquito nets otherwise we catch malaria" (M number 17, 19 years old)

Within this group women with children under five years, there are some who think that all age groups should use the nets:

"We all need to be protected from malaria, but I can first give a net to the children, then to the adults." (M number 04, 19 years old)

"Everyone can use it, even pregnant women, children, and adults, to avoid malaria" (M number 23, 21 years old)

3. Myths, taboos and beliefs related to the use of mosquito nets

There were no myths or taboos among **pregnant women** (21/24) in the community, on the contrary they were motivated to use mosquito nets to prevent malaria by their community leaders:

"they said we should take care of the mosquito net, it is forbidden to use it to cover veggie gardens or for fishing." (PW number 17, 23 years old)

"We only heard that we should use mosquito nets to protect children and not to protect gardens" (PW number 13, 19 years old)

Only a few pregnant women (3/24) reported hearing that mosquito nets increased the number of mosquitoes and thus led to more diseases:

"No, I haven't heard a lot of things about the use of mosquito nets. what I have heard sometimes is that the net may cause infections or increase the presence of insects when used." (PW number 03, 18 years old)

"I've never heard anything about myths, I just know that they say the net has diseases and we can't use it, but I know they're fooling us and they say that we only cover ourselves so the mosquito doesn't get in, it's not because the mosquito net kills mosquitoes." (PW number 02, 32 years old)

There have never been any myths or taboos associated with mosquito nets among **women with children under 5 years of age**:

"I never heard anything about the mosquito net, apart from knowing the importance of the mosquito net" (M number 13, 20 years old)

"In the community we have never heard information or myths about the use of mosquito nets, I have never heard anything strange about mosquito nets" (M number 02, 18 years old)

4. Personal barriers to the use of mosquito nets

In insects impregnated in mosquito nets, almost all **pregnant women** report experiencing adverse effects such as allergies, phobias, and feeling of rising temperatures. According to them, they had such effects because the mosquito net hadn't been washed before use and was not stretched properly:

"Before I knew that the net had to be washed first, once I took the net without washing it and that medicine that is in the nets caused me allergies and I was breathing badly, but I didn't know what to do, but a brother also appeared and I told him that I would not use the mosquito net anymore because it created allergies and he said not to use the net before washing and letting it air outside and I did that. After that, I see the net helps a lot" (PW number 09, 40 years old)

"Even if I don't like it I still use it so I don't die of malaria. The net creates allergies, gives me pimples and heat up a lot…" (PW number 05, 37 years old)

Pregnant women (4/24) also reported breathing difficulties and allergies:

"women always say that [breathing and pimples] can be cause by mosquito nets, and can be worse when the person is pregnant." (PW number 17, 23 years old)

Among **women with children under 5** (13/24), more than half said they had never experienced any adverse reactions to the insecticide-impregnated net:

"Nothing has ever happened to me. The first time I used the mosquito net it made my face itch a lot, but after a while it passed, I didn't need to go to the hospital and it didn't take long" (M number 06, 36 years old)

"Nothing strange has ever happened to me with the net, I always like the net and I don't feel good outside the net anymore" (M number 22, 23 years old)

Four out of 24 women with children under 5 reported adverse events while using the mosquito net, including increased body temperature, allergies, and suffocation:

"When I don't wash the net it gives me pimples, but when I wash it doesn't give me pimples. Also, the net can heat up, but it is alright because it does not allow mosquitoes to enter" (M number 19, 27 years old)

"Nothing, nothing has ever happened to me because I like the mosquito net, but sometimes it happened that I got inside the mosquito net and I didn't feel well, but I never stopped using it […], I didn't breathe well." (M number 02, 18 years old)

Two women with children under 5 said that entering and exiting the mosquito net was uncomfortable in the event of necessity:

"…hiiiiiii! For me it makes it very difficult to go out to pee and go back to sleep, sometimes it bothers me because if I don't close it well and mosquitoes get in…" (M number 17, 25 years old)

Additionally, some of the women with children under five years of age report not using mosquito nets regularly because they are using other malaria prevention methods:

"I use mafurreira [Trichilia emetic] tree leaves for mosquitoes" (M number 20, 18 years old)

"…to protect my health, I use baygon [insecticide]" (M number 03, 19 years old)

5. Community barriers to the use of mosquito nets

During the interview, 16 out of 24 **pregnant women** identified allergies, feelings of suffocation, and rising temperatures as barriers to neighbours and acquaintances using mosquito nets effectively:

"Some people say that they prefer to use dragão [insecticide] *because the mosquito net is uncomfortable, and they are unable to breathe well"* (PW number 10, 42 years old)

"Many people speak ill of mosquito nets, for example, they say that they can't breathe well, it gives them pimples and they prefer not to use the mosquito net and I always convince them that one day they will get used to it" (PW number 05, 37 years old)

The perception that mosquito nets do not prevent malaria, and alcoholic habits, were listed as additional barriers by two pregnant women. Moreover, these barriers contribute to the incorrect use of nets (e.g. for fishing):

“The people who don’t like mosquito nets are usually those who abuse alcohol, so when they come back home drunk, they are unable to stretch and use the net and get bitten by mosquitoes. Others also say that the nets cause allergies and asphyxiate them, but if they washed the net before using it these effects would be reduced.” (PW number 11, 33 years old)

“People say they don't like using mosquito nets because they don't have mosquitoes and don't see the importance of using them. Other people drink too much and forget to use them or take their nets to go fishing.” (PW number 13, 19 years old)

Some pregnant women claim that some people refuse to use nets because they aren't concerned about their health, have allergies, or have suffocated. Consequently, the nets are used to cover hen houses and other items:

“For me, I don't know how to explain it, but I can say some people are careless about their health and others just don’t like using the mosquito net." (PW number 24, 20 years old)

“Other people use the mosquito net as a hen house for chickens and ducks. Some neighbours say that the net heats up and tends to cause pimples and because of that they prefer to use dragão [insecticide]” (PW number 22, 21 years old)

According to the majority of **women with children under 5 years** (22/24) neighbours and acquaintances have complained of allergies and suffocation caused by the nets:

“Other people say that when they stretch the mosquito net, they have allergies and a lot of pimples come out and others say that they don't sleep well in the mosquito net because they can’t breathe well.” (M number 13, 44 years old)

“Others say that it makes the skin itchy, but I always advise them not to stop using it” (M number 11, 37 years old)

According to two women with children under 5 years of age, acquaintances who used mosquito nets without washing them reported that they had flu:

“They just say it causes allergies and also causes you to have the flu when you use it without washing it, they might say other things but I don’t know” (M number 04, 19 years old)

6. Preferential use of mosquito net by seasons

Most **pregnant women** (13/24) prefer to use mosquito nets during summer because mosquitoes are absent during winter (see table 4):

“…I use the net more in hot weather because it's the time with a lot of mosquitoes due to the stagnant water we find there in our areas and when it gets dark the mosquitoes increase. In winter, I don't use mosquito nets much because I use the blanket more so I don't get bitten by mosquitoes” (PW number 22, 21 years old)

In one pregnant woman's experience, mosquito nets don't allow for enough air circulation in the winter, leading to discomfort:

“I use the mosquito net more in the summer than in the winter. The air does not come out because of the cold and this causes me discomfort.” (PW number 06, 20 years old)

More than half (17/24) of **women with children under 5 years** use mosquito nets throughout the year (see table 4):

"Very much in the summer, because there are a lot of mosquitoes, but as the weather has changed, even this winter there are a lot of mosquitoes, but I always used it even though it was winter" (M number 22, 23 years old)

Table 4: Preferential use of mosquito net by season

Seasons	Pregnant women (24) (n)	Women with children under 5 years (24) (n)
Only in summer	14	3
Throughout the year	10	17

7. ***Key messages from healthcare workers during the distribution of mosquito nets at the ANC***

Approximately half of the **pregnant women** (11/24) reported only receiving the mosquito net, without any instructions on how to care for it:

"Here at the hospital they just gave me the mosquito net and didn't say anything else" (PW number 04, 36 years old)

"Here at the hospital they didn't say anything else, they just gave me the net and told me to use it" (PW number 08, 41 years old)

In the ANC, five pregnant women were told that before using the mosquito net, it should be washed, laid in the sun for the insecticide to evaporate:

" The distributors said that before I use the mosquito net I should wash the net, let it out in the sun so that the medicine on it disappears. In very hot conditions, it should be left to cool down, soak up the sun for a period of two days. I was told to wash it in the basin, using detergent soap" (PW number 08, 41 years old)

"They said we should spread the net in the sun so that medicine evaporates and we don't itch" (PW number 19, 38 years old)

A further five pregnant women were instructed not only to wash the mosquito net before use, but also to refrain from using it for fishing and even to cover their flowerbeds:

"they said that the nets are for preventing mosquitoes so we don't have malaria, they are not to be used to cover the flower beds" (PW number 14, 19 years old)

Among women with children under 5 years, most (17/24) said they were only instructed to use nets to prevent mosquito bites, but not how to care for them:

"Here, they only told me that I should use the mosquito net to prevent malaria, otherwise this person inside my belly could come out with malaria or other diseases, and they didn't say anything else." (M number 18, 22 years old)

"here at the hospital they didn't tell us anything, they just gave us the net, we were told most things by those who distributed the net in the communities." (M number 03, 19 years old)

Four **women with children under 5 years** were informed at the ANC that they should wash and stretch the net in the shade to reduce the insecticide adverse effects:

“Here at the hospital they told me to wash the net and stretch it out to avoid mosquito bites. They said I should wash it with soap and hang it in the shade, but I can’t remember for how long.” (M number 08, 34 years old)

“…they told me to spread it out in the shade to reduce the medicine, they didn't tell me to wash it.” (M number 04, 19 years old)

8. Key messages from healthcare workers during the large-scale distribution of mosquito nets

Half of the **pregnant women** (12/24) said that they were not informed about how to take care of the mosquito net:

“They didn't say anything they just gave us the mosquito nets” (PW number 13, 19 years old)

“…they said we should use the net to prevent malaria and they didn’t say anything else” (PW number 02, 18 years old)

A few pregnant women (5/24) were instructed to wash the net in powder detergent and stretch it in the sun to prevent adverse effects from the insecticide:

“they said that we should wash it with OMO before using it so that the medicine in the net doesn’t cause side effects and that we could also lay it out in the sun” (PW number 07, 23 years old)

“Those who distributed the nets in the community said that they were not for washing, but for stretching out in the sun so that the medicine would not harm me” (PW number 22, 21 years old)

Four pregnant women were told to use the nets for malaria prevention and not for fishing or covering flower beds:

“They said they don't want to see the nets stretched out to protect the gardens and they also don't want to see us use the nets for fishing in the lake and they didn't say anything else” (PW number 20, 32 years old)

“They said that the nets were to be used to prevent mosquitoes so we wouldn’t get malaria, they weren’t to be used to cover the flowerbeds” (PW number 17, 23 years old)

The majority of **women with children under 5 years** (15/24) reported that the health professionals only told them mosquito nets were for preventing mosquito bites. No instructions were given about how to maintain the nets:

"They said I should keep myself inside the net and they didn't tell me anything else"(M number 01, 27 years old)

“They didn't say anything they just gave us the mosquito nets and didn't explain” (M number 21, 24 years old)

In some cases, women with children under 5 years were also advised to wash the nets with a detergent and lay them out in the sun; in others, they were simply instructed to lay them out in the sun or in the shade without specifying a period of time:

"they said we should hang out the net in the shade, the time/duration I don't know anymore. And that we shouldn't spread the net in the sun either and I don't remember the rest anymore" (M number 18, 22 years old)

"they said to wash it with detergent soap, lay it out in the sun to dry and then stretch it indoors." (M number 15, 29 years old)

9. ***Facilitating factors that may influence the use of mosquito nets in pregnant women and women with children under 5 years***

It is expected that the women should be able to explain everything about mosquito net care before they use them if the health professionals provided key messages about mosquito nets during mass distribution and at the ANC. This would prevent the women from becoming discouraged by side effects which may influence them to stop using mosquito nets:

"even now they gave us the net, but they didn't explain to me what I should do before starting to use it, so the Ministry of Health should review this and tell the nurses as well as the distributors of mosquito nets in the community to give a good explanation of the use of mosquito nets. Others open it and use it right away and suffer from allergies, so they may decide not to use the mosquito net anymore after this" (PW number 10, 37 years old)

There were some participants who believed that the ministry of health shouldn't just distribute mosquito nets. Additionally, it should monitor mosquito net usage and deliver health talks in schools and communities:

"To make things easier and encourage people to use the mosquito net properly, after giving us the nets, they should talk about it in schools and teach us how to use it." (PW number 15, 42 years old)

"Health authorities should also use schools, our local chiefs so that they can teach us how to use to mosquito nets correctly, and monitor the use in the community so that people are not using it in the mangroves and for fishing." (M number 20, 18 years old)

Other participants reported that the distribution of mosquito nets by the government is already an important facilitator:

"For me and my family, it is important that the ministry of health continues to distribute mosquito nets" (M number 07, 47 years old)

"The government must always distribute mosquito nets because, for example, they are very expensive for the money we make and it can happen that others don't have the money to buy mosquito nets and die of malaria" (PW number 10, 37 years old)

Discussion

Knowledge about the correct use of mosquito netting

In this study, it was found that more than 50% of pregnant women and mothers with children under five years of age knew mosquito nets prevent mosquito bites, and, consequently, malaria. In studies conducted in African countries (South Africa and Ethiopia), pregnant women and mothers with young children have stated that ITNs always serve as a physical barrier between

them and mosquitoes, reducing the risk of mosquito bites. According to the same studies, ITNs also disorient or kill resting mosquitoes by their repellent effect(8,9).

Considering that participants might be reluctant to give honest answers about mosquito nets, participants were also asked about the opinions of their closest friends. Several interviewees mentioned that their neighbours used the nets to fish and protect flowerbeds due to the smell and increased heat. Those findings are consistent with those found in a study conducted in Malawi, which concluded that respondents did not reveal the truth for fear that the government would paralyze net distribution. Researchers in that study sought to find out what other people knew about ITNs and how they were being used. Answers varied, but the main one was that participants believed the nets could be sold or used for fishing in order to increase family income due to drought and food insecurity (10).

Knowledge about the use of mosquito nets in vulnerable groups

Among the interviewees who were knowledgeable about vulnerable groups for malaria and which groups should be prioritized for mosquito nets, pregnant women and young children were identified as the most vulnerable groups in need of special protection such as continuous provision of mosquito nets. These results corroborate the results of a study carried out in Ethiopia that, when using ITNs, the population understands how important it is to protect children and pregnant women(11).

Despite the fact that they may live with children or pregnant women who are easily susceptible to malaria, some women understand that all age groups need equal attention. In Madagascar, a study concluded that other groups should also be protected to prevent them from contaminating vulnerable groups living around them(12). Researchers in the Central region of Mozambique found that school-aged children are at greater risk of malaria infection and severe disease (13).

Myths, taboos and beliefs related to the use of mosquito nets

It was found that some participants believed the use of mosquito nets would increase the population of insects and diseases. According to a study conducted in Ethiopia, respondents believed that ITNs breed bed bugs (small parasitic insects that feed exclusively on humans and domestic animals' blood)(8).

Personal barriers to the use of mosquito nets

According to the study, many mothers with children under five do not encounter any difficulties while using the ITN and no longer feel comfortable sleeping without it. While they mention allergies, increased temperatures and suffocation as direct challenges for neighbours or acquaintances who don't use the nets or don't use them regularly. Unlike the pregnant women, they responded openly and said that, both for themselves, as well as for their neighbours and acquaintances, they had already experienced allergies, suffocation, and increased temperatures, especially during the summer, which led to using ITNs for fishing and protecting flowerbeds and yards.

Two studies conducted in Ghana and Rwanda found similar results. Based on these studies, all respondents rated heat and discomfort under nets as major barriers to consistent use(14). The most common reasons respondents gave for not using ITNs were the feeling of warmth and itching when using them(15). In addition to physical discomfort, concerns about chemicals impregnated in the ITN are also common reasons not to use one (16).

According to a study conducted in Rwanda, respondents were not using ITNs because they simply didn't have them, contrary to the findings of this study (17). Since 2011, Mozambique has been implementing massive mosquito net distribution campaigns every three years. The lack of mosquitoes and insufficient ITNs have been reported by several studies in central Mozambique as reasons for not using ITNs(13).

It was also stated that mothers whose children were under five were uncomfortable leaving and entering the net at night, but that at some point they became comfortable and didn't prioritize the use of the net because they had already sprayed their homes or used other methods to repel mosquitoes, such as plants.

A study in Ghana found that there were other reasons why ITNs were not used. There are a number of reasons for this, including the use of herbs, grass, burning orange peels, and mafur tree peels as alternatives. It is believed that these peels repel mosquitoes effectively. Some people believed that their surroundings did not have any mosquitoes, while others said they sprayed their rooms, so there were no mosquitoes and they did not need ITNs (15). In addition, the difficulty of getting in and out of the RTI at night was cited as a barrier to utilization. Sleeping under the net restricted some participants' movement during the night and made it difficult for them to get in and out of bed (14).

Preferential use of mosquito net by seasons

According to the present study, pregnant women use ITNs in summer because there are more mosquitoes during this period than during winter. The results of two studies, one in Thai-Myanmar and the other in Madagascar, indicate that women use ITNs more in summer because they believe there are no mosquitoes in winter (12,18). These results corroborate those found in the present study, in contrast to a study carried out in Nigeria that concluded pregnant women do not use the ITN in the summer because it is uncomfortable given the rise in temperature in the summer than in winter (19). Several mothers with children under five years of age reported using the ITN throughout the year, as they observed mosquitoes during both seasons (20).

Key messages in prenatal consultations and mass ITN distribution campaigns

Results of the present study show that participants were informed effectively about the use of mosquito nets for malaria prevention during ANC consultations and mass distribution. However, the care for mosquito nets before use was not effectively transmitted. In this scenario, it is possible that mosquito nets were misused or were not used properly, resulting in adverse reactions to the insecticide, which can cause strong allergies. According to studies, radio/TV advertisements and key messages from national leaders influenced women to use ITNs, despite not receiving appropriate messages in health facilities (8,15).

During prenatal consultations and during the campaign, interviewees were told they should wash mosquito nets with powdered detergent and lay them out in the sun before using them, but this is incorrect information given by health care providers. The detergent removes all the insecticide impregnated in the mosquito net, so it becomes merely a physical barrier against mosquitoes, reducing its effectiveness.

According to a study with health professionals in eight units, they generally provided comprehensive information about ITNs before their use. The information provided included tips on how to prepare the ITNs before use, how to dry them in the shade for 24 hours to avoid

allergies caused by the chemicals used to treat them, how to prevent malaria during pregnancy, and how to protect the body from mosquito bites by using ITNs. When expecting mothers need to be outdoors for long periods of time during the night, they were advised to wear long-sleeved dresses to prevent mosquito bites. A dialogue sometimes occurred between pregnant women who were reluctant to receive ITNs and health professionals at the ANC, where health professionals attempted to ease their fears and misconceptions about ITNs causing heat and itching in the body and eyes (15).

Facilitating factors that may influence the use of mosquito nets in pregnant women and women with children under 5 years

As a facilitator for the efficient use of ITNs, the study highlighted the need for sufficient and accurate information during the distribution of ITNs at the ANC and integrated (multisectoral) post-campaign awareness events. In Nigeria, Francis Ugwu (2023) found that awareness after the distribution of ITNs was a determining factor for ITN use (21). According to a study conducted in a school context, ITNs should continue to be distributed for free in low-income countries and students should be mobilized to promote their use (22). In Gaza, a study found that community groups can help disseminate key messages and facilitate the use of ITN (23).

Limitations

The study had some notable limitations. Women were hesitant to participate in the study, because participation meant staying for a longer period than initially planned in the health facility. To minimize this limitation, during the health talks in the waiting rooms the women were informed beforehand about the study and its procedures. Observer bias was an additional limitation and to address this the investigators used follow up questions whenever necessary. Women were also hesitant about participating in the study in fear of suffering retaliation and being excluded from future ITNs distribution campaigns. These fears were also addressed during the health talks and before the interviews, to ensure they understood that their responses would not influence current or future healthcare provision and that they were free to stop their participation anytime they felt uncomfortable.

Conclusion

Despite extensive distribution of mosquito nets at health facilities and in communities, there still exists a large knowledge gap amongst the participants and their communities on the correct use of the mosquito nets. There are still reports of misuse of the mosquito nets in the community as a result of side effects from incorrect use and misinformation. This may explain in part the persistent high incidence of malaria in these districts. It is crucial for the health professionals, ministry of health and its partners to review current strategies to include extensive community engagement (community awareness campaigns) and regular monitoring of correct use of ITNs (not just coverage).

Acknowledgments

The authors would like to thank the women who participated in the study, the data collectors and the health professionals in the facilities selected for the study.

References

1. Ejigu BA. Geostatistical analysis and mapping of malaria risk in children of Mozambique. PLOS ONE. 2020 Nov 9;15(11):e0241680.

2. OMS Africa. OMS | Escritório Regional para a África. 2023 [cited 2023 Jul 18]. In Mozambique, household screening keeps families malaria-free. Available from: https://www.afro.who.int/countries/mozambique/news/mozambique-household-screening-keeps-families-malaria-free

3. WHO. World malaria report 2022 [Internet]. [cited 2023 Jul 18]. Available from: https://www.who.int/teams/global-malaria-programme/reports/world-malaria-report-2022

4. Programa Nacional de Controlo da Malária. Plano estratégico da malária 2017–2022. Moçambique: Ministério da Saúde; 2017.

5. INS. Inquérito Nacional sobre Indicadores de Malária (IIM) 2018 [Internet]. Moçambique; 2019. Available from: https://dhsprogram.com/pubs/pdf/MIS33/MIS33.pdf

6. Misau, INE, ICF. Inquérito de Indicadores de Imunização, Malária e HIV/SIDA em Moçambique (IMASIDA) 2015. MISAU [Internet]. 2018 [cited 2021 Feb 25]; Available from: https://dhsprogram.com/publications/publication-ais12-ais-final-reports.cfm

7. INE. Folheto estatístico distrital, 3–4. Moçambique; 2020.

8. Admasie A, Zemba A, Paulos W. Insecticide-Treated Nets Utilization and Associated Factors among under-5 Years Old Children in Mirab-Abaya District, Gamo-Gofa Zone, Ethiopia. Front Public Health. 2018;6:7.

9. Kanyangarara M, Hamapumbu H, Mamini E, Lupiya J, Stevenson JC, Mharakurwa S, et al. Malaria knowledge and bed net use in three transmission settings in southern Africa. Malaria Journal. 2018 Jan 19;17(1):41.

10. Berthe S, Harvey SA, Lynch M, Koenker H, Jumbe V, Kaunda-Khangamwa B, et al. Poverty and food security: drivers of insecticide-treated mosquito net misuse in Malawi. Malaria Journal. 2019 Sep 18;18(1):320.

11. Yhdego TG, Gardew AD, Yifat FT. Malaria prevalence, knowledge and associated factors among household heads in Maygaba town, Ethiopia. PLOS Glob Public Health. 2022;2(3):e0000071.

12. Njatosoa AF, Mattern C, Pourette D, Kesteman T, Rakotomanana E, Rahaivondrafahitra B, et al. Family, social and cultural determinants of long-lasting insecticidal net (LLIN) use in Madagascar: secondary analysis of three qualitative studies focused on children aged 5–15 years. Malaria Journal. 2021 Mar 26;20(1):168.

13. Scott J, Kanyangarara M, Nhama A, Macete E, Moss WJ, Saute F. Factors associated with use of insecticide-treated net for malaria prevention in Manica District, Mozambique: a community-based cross-sectional survey. Malaria Journal. 2021 Apr 27;20(1):200.

14. Ahorlu CS, Adongo P, Koenker H, Zigirumugabe S, Sika-Bright S, Koka E, et al. Understanding the gap between access and use: a qualitative study on barriers and facilitators to insecticide-treated net use in Ghana. Malaria Journal. 2019 Dec 12;18(1):417.

15. Aberese-Ako M, Magnussen P, Ampofo GD, Tagbor H. Health system, socio-cultural, economic, environmental and individual factors influencing bed net use in the prevention of malaria in pregnancy in two Ghanaian regions. Malaria Journal. 2019 Nov 12;18(1):363.

16. Yitayew AE, Enyew HD, Goshu YA. Utilization and Associated Factors of Insecticide Treated Bed Net among Pregnant Women Attending Antenatal Clinic of Addis Zemen Hospital, North-Western Ethiopia: An Institutional Based Study. Malaria Research and Treatment. 2018 Dec 24;2018:e3647184.

17. Fabens IN. Determinants for Exposure to Mosquitoes and Bed Net Use Behavior in the Context of the New Nets Project in Rwanda [Internet]. University of Washington ProQuest Dissertations Publishing; 2021 [cited 2023 Jul 21]. Available from: http://hdl.handle.net/1773/47226

18. Pooseesod K, Parker DM, Meemon N, Lawpoolsri S, Singhasivanon P, Sattabongkot J, et al. Ownership and utilization of bed nets and reasons for use or non-use of bed nets among community members at risk of malaria along the Thai-Myanmar border. Malaria Journal. 2021 Jul 6;20(1):305.

19. Bhalla D, Cleenewerck L, Okorafor Kalu S, Abubakar Gulma K. Malaria Prevention Measures among Pregnant Women: A Population-Based Survey in Nnewi, Nigeria. ScientificWorldJournal. 2019;2019:6402947.

20. Tassew A, Hopkins R, Deressa W. Factors influencing the ownership and utilization of long-lasting insecticidal nets for malaria prevention in Ethiopia. Malaria Journal. 2017 Jul 1;16(1):262.

21. Ugwu FSO. Why the World Health Organization should reconsider long lasting insecticide nets (LLIN) and indoor residual spraying (IRS) in primary mosquito/malaria control in favour of house screening. Bio-Research. 2023 Feb 4;21(1):1789–804.

22. Zeleke ZZ. Perceptions and impact of malaria health education in school children; Challenges in malaria control & elimination in Arba Minch, SNNPR, Ethiopia [Internet]. [Antwerp]: University of Antwerp; 2023 [cited 2023 Jul 18]. Available from: https://repository.uantwerpen.be/docman/irua/e7d21d/194652.pdf

23. Dako-Gyeke P, Hornuvo R, Glozah F, Asampong E, Tabong P, Nwameme A, et al. Pilot Implementation of Community Health Advocacy Teams to Improve the Effectiveness of Long-Lasting Insecticide Net Distribution through both Campaigns and Continuous Channels in Ghana: A Qualitative Study of Opportunities and Barriers to Implementation. 2023.

Annex 1 Interview guide

Click here to access/download
Supporting Information
Annex 1_Interview guide.pdf

Click here to access/download
Supporting Information
Ethics approval letter CNBS.pdf
