# Supplementary material for: Factors associated with the use of long-lasting insecticidal nets in pregnant women and mothers with children under five years of age in Gaza province, Mozambique
Source: PLOS Glob Public Health. 2024 Jan 16;4(1):e0002811. doi: 10.1371/journal.pgph.0002811 (PMC10790986; doi:10.1371/journal.pgph.0002811)
Supplement: Attachment — Submitted filename: Response to reviewers_PLOSGH.docx [file pgph.0002811.s006.docx]

**Response to reviewers**

PGPH-D-23-01427
Title: **Factors associated with the use of long-lasting insecticidal nets in pregnant women and mothers with children under five years of age in Gaza province, Mozambique**

Dear editor,

We humbly thank the reviewers for their time and valuable comments, that have helped us improve the manuscript. Please find enclosed our point by point responses to their queries. The manuscript is also attached in track changes to show the modifications, and in a clean version.

**Editor comments:**

**Please review your reference list to ensure that it is complete and correct. If you have cited papers that have been retracted, please include the rationale for doing so in the manuscript text, or remove these references and replace them with relevant current references. Any changes to the reference list should be mentioned in the rebuttal letter that accompanies your revised manuscript. If you need to cite a retracted article, indicate the article’s retracted status in the References list and also include a citation and full reference for the retraction notice.**

- We do not have not cited any retracted papers. We have added references in the revision due to the comments from reviewers. These have been added to the end of the letter.

**We have noticed that you have uploaded Supporting Information files, but you have not included a list of legends. Please add a full list of legends for your Supporting Information files after the references list.**

- Added to the manuscript below the reference list

**In the online submission form, you indicated that "The datasets analysed during the current study are available from the corresponding author on reasonable request". All PLOS journals now require all data underlying the findings described in their manuscript to be freely available to other researchers**

- The data has been uploaded as supplementary

**Reviewer #1:**

**The MS has a good question to understand the risk of pregnant ladies however few observations are seen

In Methods Residents living in both province are quite good in number why the convenient non-probabilistic sampling was done and only 48 pregnant and child bearing mother were taken in study which gives a lower confidence in the study authors should justify the sample size**

- Thank you for the observation. For a variety of reasons, we chose a convenient non-probabilistic sampling method: 1) cost concerns; the study did not have any funding, and random sampling would have been more expensive in such a large and dispersed population, 2) The study was time sensitivity to provide quick answers to a programmatic issue on the ground; 3) this was an exploratory study aimed at generating hypotheses for future studies using random sampling to be more comprehensive.

During the study, we included women who presented at the selected health centers with characteristics of interest. To determine sample size, we used theoretical or data saturation, but with the predicted minimum of 14 women in high-volume health centres and 10 in low-volume health canters. In this study, we were able to saturate the information without exceeding the minimum amount predicted.

- This was included in the manuscript under methods: Participant selection and sample size
- These limitations were included in the manuscript.

**Results As we are interested in the perception and not in the who gave the answers therefore these answers may be point wise or percent wise can be put in the MS rather using their answers. Put data in a paragraph form the answers by the respondent can be used in passive form and in percent perception etc. The result section should have such paragraphs or results and percentages as written in discussion.**

- Thank you. We have reorganized and rewritten the result section as advised.

**In discussion authors may consider writing a contentious discussion on the awareness and why the results were observed so.**

- The discussion section was revised and rewritten as advised.

**Reviewer #2:**

**The manuscript's title is somewhat unclear, as it mentions mosquito nets, while a portion of the manuscript specifically focuses on LLINs. Consider revising the title to reflect the broader scope of LLIN usage.**

- Thank you for the advice. The title has been revised.

**The title page of the manuscript lists authors in a different sequence than the submitted author list, and the affiliation of the corresponding author on the title page differs. These discrepancies need to be addressed.**

- Thank you for noticing this, it has been addressed. The correct order is on the manuscript.

**The use of the terms "mosquito nets" and "LLIN" appears inconsistent throughout the manuscript. It is advisable to use a consistent term for all types of nets.**

- Thank you for the advice. The term LLIN has been used throughout the manuscript.

**A thorough review of the English language and grammar is necessary, as some sentences are difficult to decipher, and punctuation issues arise, particularly in the results section.**

- The manuscript has undergone English revision. Thank you.

**The abstract format does not align with the 'Plos Global Public Health' guidelines, and it should not contain subheadings. Additionally, consider shortening the conclusion section by one or two lines.**

- Thank you, this has been addressed and the abstract has been revised as per PLOS GPH criteria.

**In the introduction section:**

**Properly format the reference "WH, 2022" in a standard referencing style.
Clarify whether Cabo Delgado is a state or district in line 3 of paragraph 2.
Replace the phrase "massive amount" with a more scientifically appropriate term, and specify the relevant authority's name in paragraph 3.**

- Thank you, reference revised. Cabo Delgado is a province, this has been added to the manuscript. Massive amount has been replaced with ‘large amounts’. The authority name has been added to the manuscript.

**Clarify whether Mozambique ranks 11th in Africa or globally in paragraph 3, line 4.
Add the prefix "national" to the malaria control strategic plan's name in paragraph 5, line 1.**

- Thank you, this has been clarified in the text (globally), and prefix added in the text.

**Consider mentioning studies from other African countries working on the same factors to highlight the existing research and identify gaps.** **Clearly articulate the gap in knowledge if studies on the mentioned factors are available.**

- Studies have been added to highlight and clearly articulate the research gap.

**The study's aim should include the country name, as mentioned in the abstract.**

- This has been added to the text.

**In the "Materials and Methods" section:**

**Revise "in depth interview process" to "in-depth interview method."**

- Thank you. The revision has been made

**Specify whether the study was conducted within a one-month or one-week time period.**

- Study was conducted in 1-week time period. This has been clarified in the methods

**Clarify whether healthcare facilities and health units are considered the same class of health centers.**

- They are the same primary health centres, however, for clarity we used health centres.

**Explain how the sample size remained consistent across both districts, considering potential data variations. It appears that the sample size was pre-determined, with interviews conducted accordingly based on the different categories.**

- Thank you for the observation. For a variety of reasons, we chose a convenient non-probabilistic sampling method: 1) cost concerns; the study did not have any funding, and random sampling would have been more expensive in such a large and dispersed population, 2) The study was time sensitivity to provide quick answers to a programmatic issue on the ground; 3) this was an exploratory study aimed at generating hypotheses for future studies using random sampling to be more comprehensive.

During the study, we included women who presented at the selected health centers with characteristics of interest. To determine sample size, we used theoretical or data saturation, but with the predicted minimum of 14 women in high-volume health centres and 10 in low-volume health canters. In this study, we were able to saturate the information without exceeding the minimum amount predicted.

- This was included in the manuscript under methods: Participant selection and sample size
- These limitations were included in the manuscript.

**Clarify whether the interviews were conducted individually or in the presence of family members.**

- Thank you. This has been clarified in the text. They were conducted individually.

**Provide information on who translated the past questions and responses when the native language was used.**

- Translations were carried out by one of the study surveyors and double checked by the principal investigator. This has been added to the manuscript.

**Specify how many individuals were contacted and how many agreed to participate in the study.**

- As women presented at the consultation and an investigator was available to interview them, they were recruited. If all investigators were conducting interviews, some women might have come for consultation and left without being interviewed. A daily average of 10 -15 pregnant women and 18-25 mothers with children under 5 years attend the health center for consultation.

**Elaborate on the timeline and individuals responsible for data analysis.**

- Analysis, interpretation of the data and elaboration of the final report were conducted between July 2022 and February 2023.

**If the interview module used was developed in 2021 and used in the 2022 study, please reference the source of the interview module if it was utilized in a previous study.**

- The interview guide was study specific and has not been utilized in a previous study.

**In the "Results" section:**

**Address the discrepancy between the occupation categories and the module questions, particularly regarding the "stay at home" and "white stick whites" categories. Provide a brief overview of the data presentation before presenting the actual data. Consider including a table of themes and subthemes to enhance reader comprehension. In paragraph 2, line 3, discuss the relevance of "Nets to fish and protect flower beds due to smell and increased heat" in the results section, as it was not previously mentioned.**

- The results section was revised and rewritten as advised.

**Discuss the study results in the context of previous research conducted on the same cohort in African and global populations.**

- The discussion section was revised and rewritten as advised.

**The following references were added during the revisions:**

- Wetzler EA, Park C, Arroz JAH, Chande M, Mussambala F, Candrinho B. Impact of mass distribution of insecticide-treated nets in Mozambique, 2012 to 2025: Estimates of child lives saved using the Lives Saved Tool. PLOS Global Public Health. 2022 Apr 26;2(4):e0000248.
- Berthe S, Harvey SA, Lynch M, Koenker H, Jumbe V, Kaunda-Khangamwa B, et al. Poverty and food security: drivers of insecticide-treated mosquito net misuse in Malawi. Malaria Journal. 2019 Sep 18;18(1):320.
- Jones BL, Unsworth RKF. The perverse fisheries consequences of mosquito net malaria prophylaxis in East Africa. Ambio. 2020 Jul 1;49(7):1257–67.
- Gutiérrez-Jara JP, Vogt-Geisse K, Cabrera M. Collateral Effects of Insecticide-Treated Nets on Human and Environmental Safety in an Epidemiological Model for Malaria with Human Risk Perception. Int J Environ Res Public Health. 2022 Dec 6;19(23):16327.
- Kumar R, Farzeen M, Hafeez A, Achakzai BK, Vankwani M, Lal M, et al. Effectiveness of a health education intervention on the use of long-lasting insecticidal nets for the prevention of malaria in pregnant women of Pakistan: a quasi-experimental study. Malaria Journal. 2020 Jun 29;19(1):232.

We hope that we have addressed their points satisfactorily and appreciate the opportunity to submit the new manuscript according to reviewers’ comments. All authors have seen and approved the revised version. We look forward to hearing from you soon.

Kind regards,

Isabelle Munyangaju

(Corresponding author)
