# Supplementary material for: Factors associated with the use of long-lasting insecticidal nets in pregnant women and mothers with children under five years of age in Gaza province, Mozambique
Source: PLOS Glob Public Health. 2024 Jan 16;4(1):e0002811. doi: 10.1371/journal.pgph.0002811 (PMC10790986; doi:10.1371/journal.pgph.0002811)
Supplement: Attachment — Submitted filename: Response to reviewers_PLOSGH_RV2.docx [file pgph.0002811.s008.docx]

**Response to reviewers**

PGPH-D-23-01427
Title: **Factors associated with the use of long-lasting insecticidal nets in pregnant women and mothers with children under five years of age in Gaza province, Mozambique _ Revision 2**

Dear editor,

We appreciate the reviewer’s comments, please find enclosed our point by point responses to them. The manuscript is also attached in track changes to show the modifications, and in a clean version.

**Reviewer #1:**

**What is high volume PHC and low volume PHC the disease is malaria to be studied is the case quantity was taken as criteria or number of persons visiting**

- Thank you for the observation.
- This clarification was added to the text: ‘*The number of monthly mother and child health (MCH) consultations was used to define volume, a high volume PHC would have ≥ 220 MCH consultations per month. Low volume PHC would have <150 MCH consultations per month.*’

**How does the it affects the cost if more samples were taken as it was only in-depth interview only and was cross-sectional only?**

- Thank you. The costs we are talking about are the additional personnel costs for the additional time, rental of recording devices, transport costs).
- We have added this to the manuscript for clarification to the reader.

We hope that we have addressed their points satisfactorily and appreciate the opportunity to submit the new manuscript according to reviewers’ comments. All authors have seen and approved the revised version. We look forward to hearing from you soon.

Kind regards,

Isabelle Munyangaju

(Corresponding author)
